# Modeling of stringent-response reflects nutrient stress induced growth impairment and essential amino acids in different *Staphylococcus aureus* mutants

**DOI:** 10.1038/s41598-021-88646-1

**Published:** 2021-05-06

**Authors:** Christof Audretsch, Fabio Gratani, Christiane Wolz, Thomas Dandekar

**Affiliations:** 1 Institut Für Med. Mikrobiologie und Hygiene, Elfriede-Aulhorn-Straße 6, 72076 Tübingen, Germany; 2 Department of Bioinformatics, Biocenter, University of Würzburg, Am Hubland, 97074 Würzburg, Germany; 3 EMBL Heidelberg, Bioinformatics, Meyerhofstraße 1, 69117 Heidelberg, Germany; 4 Present Address: Quantitative Proteomics and Proteome Center Tuebingen, Interfaculty Institute for Cell Biology, University of Tübingen, Tübingen, Germany

**Keywords:** Cellular signalling networks, Bacteriology

## Abstract

*Stapylococcus aureus* colonises the nose of healthy individuals but can also cause a wide range of infections. Amino acid (AA) synthesis and their availability is crucial to adapt to conditions encountered in vivo. Most *S. aureus* genomes comprise all genes required for AA biosynthesis. Nevertheless, different strains require specific sets of AAs for growth. In this study we show that regulation inactivates pathways under certain conditions which result in these observed auxotrophies. We analyzed in vitro and modeled in silico in a Boolean semiquantitative model (195 nodes, 320 edges) the regulatory impact of stringent response (SR) on AA requirement in *S. aureus* HG001 (wild-type) and in mutant strains lacking the metabolic regulators RSH*,* CodY and CcpA, respectively. Growth in medium lacking single AAs was analyzed. Results correlated qualitatively to the in silico predictions of the final model in 92% and quantitatively in 81%. Remaining gaps in our knowledge are evaluated and discussed. This in silico model is made fully available and explains how integration of different inputs is achieved in SR and AA metabolism of *S. aureus*. The in vitro data and in silico modeling stress the role of SR and central regulators such as CodY for AA metabolisms in *S. aureus*.

## Introduction

*Staphylococcus aureus* is often part of the natural flora without causing infections (commensal lifestyle)^1^. Yet it is also one of the most important pathogens causing a wide range of diseases in almost any region or organ of the body ranging from skin and wound infection to endocarditis or osteomyelitis^2^. The high virulence potential of *S. aureus* is also due to its genomic variability and capability to adapt to different environmental conditions^3,4^.

The observation that *S. aureus* requires many amino acids (AAs) for growth despite the presence of all essential biosynthesis gene clusters indicates that auxotrophy might in large extent be due to regulatory circuits. At least three global regulatory systems, the stringent response (SR) and the transcription factors CodY and CcpA, contribute to the expression of amino acid metabolic genes. A comprehensive understanding of the effects of these regulators on AA synthesis requires the integration of all sensed signals and downstream effects.

AA limitation leads to activation of the (p)ppGpp synthetase domain of the RSH enzyme (RelA/SpoT homolog)^5^. *S. aureus* also possesses two small (p)ppGpp synthetases, RelP and RelQ. However, they are non-responsive towards AA limitation but likely contribute to a basal level of (p)ppGpp under non-stressed conditions^6^. In Firmicutes, (p)ppGpp synthesis is accompanied by a severe drop of the intracellular GTP level which leads to downregulation of processes like ribosome biogenesis and translation and causes de-repression of CodY target genes^6,7^. The global regulator CodY controls the expression of a large number of metabolism and virulence genes, including many genes involved in AA biosynthesis and transport, in response to the availability of GTP and the branched-chain amino acids isoleucine, leucine, and valine^8,9^. CcpA, the master regulator of carbon catabolite repression, is another known modulator of AA metabolism in *S. aureus*. Under conditions of glucose availability, CcpA activates key enzymes of the glucose metabolism which provides necessary intermediates for AA biosynthesis. Moreover, CcpA directly regulates genes involved in arginine and proline biosynthesis^10,11^ and in amino acid catabolism, thereby linking utilization of alternative carbon sources to glucose availability^12,13^.

Hence the goal of this work is to model the regulation of AA metabolism, including major regulatory components (stringent response, CodY and CcpA), in a Boolean network and to validate the model based on the growth behavior of the *S. aureus* HG001 parental strain and isogenic mutants defective in *RSH*, *codY* and *ccpA*, respectively. Our approach allows to (i) gain a better understanding of the regulatory processes, (ii) develop a useful in *silico* tool for hypothesis testing and (iii) evaluate the essential AA in different *S. aureus* strains. Moreover, this may be used for development of a new anti-staphylococcal treatment strategy.

## Methods

### Strains and growth conditions

Bacterial strains are listed in Table 1. HG001 *ccpA* and (p)ppGpp0-21 (the (p)ppGpp0 codY mutant) were obtained by transduction of the *ccpA*^14^ or *codY*^15^ mutation into strain HG001 and (p)ppGpp0, respectively. *Rsh*_*syn*_ is a derivative of strain HG001 in which the synthetase domain within rsh was mutated^16^. Bacteria were grown overnight in chemically defined medium^15^ supplemented with the appropriate antibiotics (erythromycine 5 µg/ml, tetracycline 3 µg/ml). Strains were inoculated to an OD_600_ of 0.05 in fresh CDM (without antibiotics) and grown to OD_600_ of 0.5. Bacteria were pelleted and washed twice with 2 ml PBS and used to inoculate prewarmed CDM lacking single AAs in a 96 well microtiter plate to an initial OD600 of 0.05. Growth was monitored using Tecan infinite M200 Pro (Tecan Group Ltd) with data acquisition in 30 min intervals. Each strain was analyzed in triplicate.

**Table 1:** Strains used in the experiments.

StrainNr./type	Strain name	Description	Refs.
WT	HG001	rsbU restored RN1 (8325)	^55^
*codY*	HG001-21	HG001 ΔcodY::tet	^15^
c*cpA*	HG001-ccpA	HG001, ΔccpA::tet	This work
*rsh*_*Syn*_	HG001-86	HG001 *rsh*_*Syn*_ (Δ942-950nt, mutation of synthetase domain)	^16^
*relP/Q-rsh-codY*	(p)ppGpp0-21	HG001, Δrsh, Δ/relP, ΔrelQ, ΔcodY::tet	This work

### Network construction and simulation

#### Data acquisition

For setup of a first topology network of amino acid metabolism, data available from biochemical databases such as KEGG^17^ and the protein–protein interaction database STRING^18^ were used. Furthermore, data from microarray analysis of the stringent response (RSH-dependent)^16^ and other microbiological literature were considered. Judging from the genome it was determined for *S. aureus* that in principle all AA should be synthesizable^15^. Therefore, further information from GenBank and sequence annotation not only of NCTC 8325 but for unclear annotation also information from COL and Newman were considered to curate the network. Thus, all this information was used to verify the nodes and types of interactions, making sure the network comprises all the central genes, products, metabolic intermediates and enzymes as well as their interactions for stringent response and amino acid metabolism. The model represents the strain HG001 but the interactions of this model are valid and well conserved for all related strains of clade A according to^19^.

#### Network assembly

All these data sources together with expert curation by sequence and domain analysis were assembled to ultimately yield a reliable network confirmed by different data sources and analyzes that includes all biologically important functional aspects of all protein interactions. Although all major relevant interactions and proteins were considered no biophysical or biochemical details were included (e.g. affinity constants, on and off kinetics) to keep the model as simple as possible. Model connectivity was further refined by expert curation and bioinformatics analysis, for instance CodY and CcpA target genes were identified based on conserved binding motifs of metabolic genes^20^. For simulations and growth predictions, the network was constructed, visualized and made machine-readable using the software yEd-Graph Editor (v3.14.1).

For setting up the network in the yEd-Graph Editor we adhere to the following four already established rules^21^:

1. Every known, literature-reported connection concerning the SR and the AA metabolism in *S. aureus* was implemented.
2. Concerning a specific single interaction and differences in literature reports about it, the most frequently found connection type was selected when there was conflicting information. If, for example, a reaction was described several times as existing and only once as undetectable, in the synopsis the decision for the fact most frequently mentioned in the literature was made. In addition, attention was paid to the quality of the respective study on which the statement was based, so whether, for example, a connection was indirectly refuted in a study or whether it was proven in detail with all individual steps and reactions.
3. To make the simulation treatable and affordable, the model has always to be a strong simplification of the complete cellular interaction network. It has hence to focus on the network of interest, in this case the amino acid metabolism and its regulation. However, care has to be exerted not to miss important input at the rims of the subnetwork. Hence, concerning these margins or the boundaries of the model, uncertainty may arise about the importance of the node or interaction for the SR and AA metabolism of *S. aureus*. If there was at least one report available stressing its importance, we chose to implement the node at the rim and suitable interaction edges to make sure not to miss any biologically relevant component.
4. As a general rule, a biological process which cannot easily be simplified and adapted to the activating inhibiting network model we clipped strongly down to the central interactions which best reproduced the biological output correctly. We hence did not implement all available connections. Table S1 in Supplement gives detailed listing of the final network, the connections (activating or inhibiting) together with literature references.

In the AA metabolism frequently occurring AND conditions i.e. the requirement of substrates and enzymes to generate the products are implemented in the simulation by means of custom-made, developed and tested "AND-Logic-gates" (Fig. S4). These logic-gates represent the connectivity between the different regulatory inputs as shown in the figures and in accordance with the experimental data, however, these are simplified, so called “effective” models, i.e. producing the correct output but simplifying the model. The logical gates are not yet resolved at the level of concrete interactions and mediating proteins. The molecular dissection of these regulatory interactions is not yet possible with current data, because this data and thus the exact molecular interactions are not available today and because although the simulation method provides for an integration of the activating and inhibiting afferent connections, it does not provide for the representation of AND conditions. The latter limitation of the model is remedied after integrating the added logical gates into the model, however, without showing the actual interaction mechanisms, but rather the functional effects, in particular correctly predicting growth and auxotrophy.

#### Network simulation

**Figure 1:**
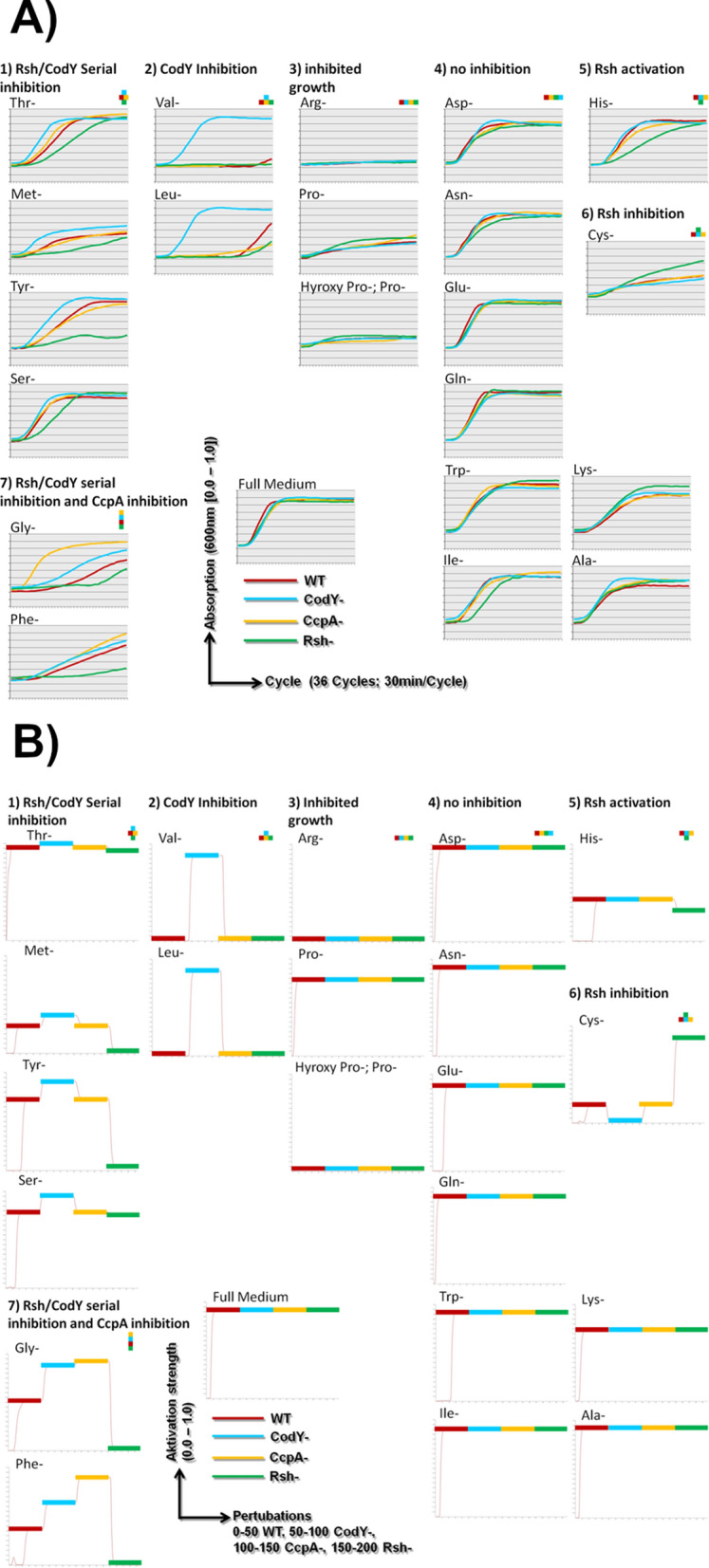
Seven patterns how AA-Synthesis depends on central nodes: Each pattern is introduced with its specific regulator pattern. Here the different in vitro growth curves (**A**) are shown (control, bottom middle: growth in complete medium) and compared to the in silico results (**B**). The phenotype of the in vitro growth as surrogate parameter for the synthesis of the deprived AA is compared to the in silico simulated AA synthesis phenotypes. Shown on the X-axis in (**A**) is the temporal course (30 min/growth step) and in (**B**) are the set perturbations (0–50 = WT, 50–100 = CodY-, 100–150 = CcpA-, 150–200 = RSH-) (Jimena version 26.02.2015; https://www.biozentrum.uni-wuerzburg.de/bioinfo/computing/jimena/^22^. The different mutants are color-coded: red = WT, blue = CodY-, light brown = CcpA-, green = RSH-. For each condition, exactly one simulation is given. The simulation reproducibly always yields the same result. For the growth experiments, on the other hand, three measurements were conducted for each growth condition. The simulations and the individual growth experiments are given in the detailed larger graphs in the supplementary material.

**Figure 2:**
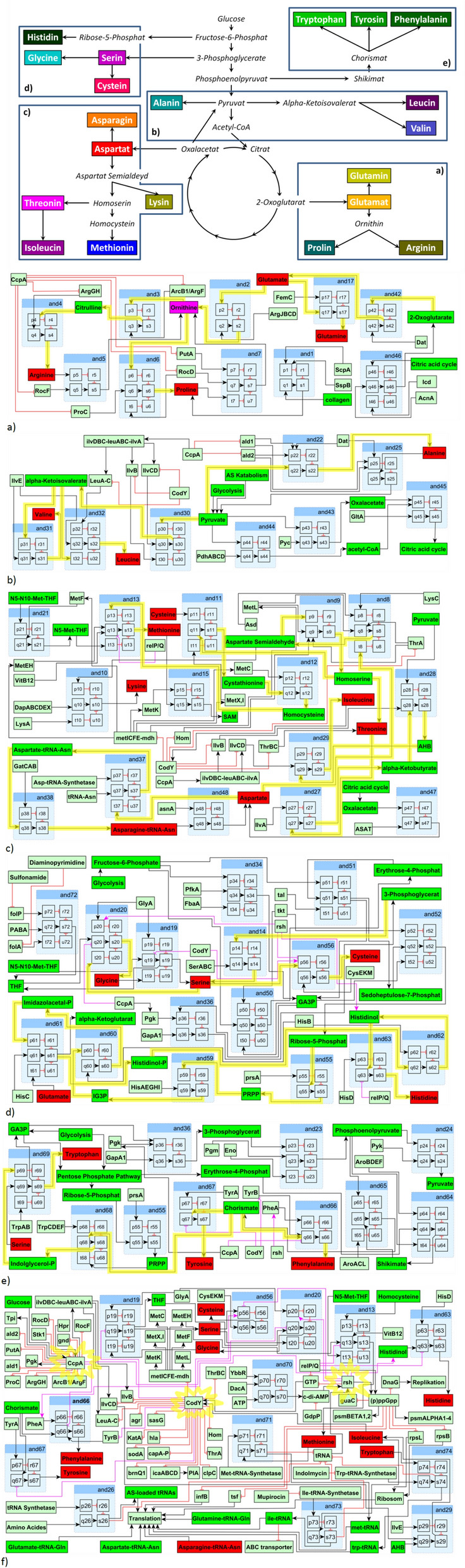
AA-Metabolism overview and sections of the Network: Top: Schematic overview display of the AA metabolism, in particular the anabolic pathways and which AA is synthesized through which pathway. "(**a**–**e**)" in the overview display describe the sections which are reflected then below in the following detailed sections of the network. Bottom: subpanel "(**f**)" shows the interconnections of the three central nodes CcpA, CodY and RSH. In the online supplement we provide the entire network as a detailed, zoomable vector graphic for close inspection of the network by the interested reader (file: Online-E-Supplement-Fig. 1E).

This graph was then loaded into Jimena^22^ a program calculating the activation or inhibition of nodes following the implemented exponential function kinetics^23^. With this our network including all the known nodes important for the AA metabolism and SR (total 195 nodes) as well as the known interactions (total 320 edges) between them was simulated (see Fig. 2 view at the main modules simulated together with a total overview; Table S1 all proteins and their interactions considered; Online E-Supplement Fig. 1E: Scalable high-resolution figure of the network). To get a constant overall activation level all input nodes with no afferent connection were activated with 0.35. The rest of the nodes were not modeled as constant in their activation but fully responding according to their inhibitory and activating input. Then Jimena was used for investigating the dynamic reaction of the network by using it for semi-quantitative simulations by reproducing the various experimental situations with in silico perturbations. For this perturbation function of the system, though the calculations of Jimena are fully continuous, the user can choose durations of certain time-steps to perturb the system. In the simulation every fifty time units, another experimental situation is simulated (see also Fig. 1b).

For these semi-quantitative simulations, the network is transformed within Jimena. The simulation calculates the activation of a single node by considering the strength of the afferent connections by taking into account the activation level of the source node by interpolating between full on and off states of the network nodes using exponential functions^23^. The strengths of the afferents are offset against each other depending on the logical connectivity (inhibitory or activating) and thus the activating or inhibiting input of the totality of afferents is determined. These e-functions approximate the kinetic parameters in a simplified manner allowing simulations that show dynamically how far a node is partially or fully activated or inhibited over time by the combination of different activating and inhibiting inputs and thus closely mirror the logical succession of events within the network.

The overall fit of the simulated activation curves modeled by the exponential functions to biological response curves is well. This works in spite of the limitation that e-functions are used, as the qualitative modeling software Jimena solves automatically the whole set of non-linear ordinary differential equations mirroring the Boolean network of the cellular signaling process. As hence all constraints have to be fulfilled at the same time, the difference between observed response curve and modeled response curve stays small^22^. This includes models of pharmacological^24^ and immune responses^25^. Moreover, we give time-units at the x-axis in our simulation outputs and results figures of the model. However, this is just for visualization, the solutions found by Jimena do not operate this way, the software calculates continuous solutions for the complete system of differential equations according to the Boolean topology modeled^22^.

The simulation provides hence a suitable dynamical model of the interactions and regulatory mechanisms in the AA synthesis in *S. aureus* as well as the influence and mechanisms of SR.

All data and the model are made available for further experimentation and investigations on SR as well as extension of the model. Though using data from *S. aureus* wild-type strain HG001, the model is easily adapted to conditions in other strains editing the network model (e.g. XML file, Cell designer editor, yEd-Graph Editor) according to the strain-specific annotation from public databases including AureoWiki^26^.

All simulations were done on a computer with Windows Home 10 (64 Bit) and Intel core I7-4700MQ CPU at 2.40 GHz and 8 GB RAM.

### In vitro–in silico comparisons

For the in silico reproduction of the in vitro experiments different time range pulses were programmed in Jimena. The growth medium was simulated by constant pulses in Jimena by activating AA present in the medium with 0.7, withdrawn AA were set to 0.0. For simulating the different knockout (KO) mutants the respective node was set to 0.0. For each condition, exactly one simulation is given. The simulation reproducibly always yields the same result. For the growth experiments, on the other hand, three measurements were conducted for each condition (see above, growth conditions).

The agreement of the in vitro experiments to alternative networks with iterative minor changes was determined for comparison and only better fitting models were accepted. In an iterative process we first incorporated further information available from literature, biochemical databases and including phylogenetic information on conserved modulatory interactions from related species and validated these networks according to our experimental data (see results, model refinement). More importantly, connections giving wrong modulatory input not leading to the observed responses were changed or removed and some initially missed connections were added. In this way, we achieved a refined network (Fig. 2 network overview; Online E-Supplement Fig. 1E as an independent file and electronic supplement offers a high-resolution view on the full network for close inspection).

We distinguish in the following a comparison regarding whether the order relation (strength of growth) was properly predicted (in silico*–*in vitro semi quantitative comparison) or whether just the growth tendency of mutant strains compared to the wild type was correctly predicted by the model (qualitative comparison).

#### In silico*–*in vitro semi quantitative comparisons

For calculation of the in vitro to in silico semi quantitative consistency, we calculated the growth performance over the whole experiment by integrating over the curve. This yields the area under the curve (AUC) which integrates over the growth and thus reflects AA synthesis performance most reliably.

Comparisons were done using categorized values. Values were categorized (weak synthesis: in silico 0.00–0.29/AUC 8–15; medium synthesis: in silico 0.30–0.69/AUC 16–21; high synthesis: in silico 0.7–1.0/AUC 22–29).

The number of categories cannot be based on empirically verifiable numbers, but must nevertheless be within a semi-quantitative comparable framework since a quantitative comparison of the in silico and in vitro data is with the current lack of kinetic and metabolite data impossible. As the number of categories increases, the quantitative character of the comparison increases. Yet when the number of categories decreases, the comparison increasingly loses its informational content. That is why after testing various possibilities the results have been divided into just three categories: weak, medium and high synthesis. This intuitively obvious division has a reasonable number of categories for a semi-quantitative comparison allowing verification or falsification of a good categorial fit of the model or not (three state prediction model). Other categorization options and their semi-quantitative consistencies were compared (Table S3 shows results for 2, 3 or 4 categories). The thresholds were determined in an analog iterative cyclic process of testing, evaluating and adjusting so that each category slot tests the predictions for this category well despite the limited data available. Depending on the question or the availability of more data, this analysis could of course be further refined. All data necessary for the evaluation of alternative categorizations (AUC or simulation data) can be found for each AA deprived medium in Table 3 (look at first line and following data).

#### In silico*–*in vitro qualitative comparisons

For the overall qualitative assessment of both in vitro and in silico, it was first determined whether the WT shows unaltered, decreased or completely inhibited growth in media lacking single AAs compared to growth in complete medium. In the next step, the relative growth of mutant strains was determined. This information was then compared between in vitro and in silico. For the optimized model, this results in a 92% agreement, which corresponds to 97 out of 105 predictions being correct.

## Results

### In vitro data: growth phenotypes of the different strains

#### Essential and modulated AA

In the *S. aureus* wild type (WT) several AA were found essential for growth, namely Arginine, Cysteine, Glycine, Leucine, Proline and Valine. Out of these Arginine and Proline are essential in all tested strains. AA deprivation of Cysteine, Methionine and Phenylalanine resulted in reduced growth in all strains. Mutations in *codY* could enhance growth in medium lacking Glycine, Methionine, Phenylalanine, Serine, Threonine, Tyrosine, Leucine and Valine. The synthesis of Glycine, Histidine, Isoleucine, Methionine, Phenylalanine, Serine, Threonine and Tyrosine is promoted by Rsh and thus reduced in the *RSH* mutant. Only in medium lacking cysteine the *rsh*_*Syn*_ mutant showed a growth advantage compared to all other strains.

Except for the effect on the synthesis of Histidine and Cysteine, effects seen in the *RSH* mutant are probably due to accompanied increase in the GTP pool and CodY de-repression of AA biosynthesis genes. Glycine and Phenylalanine are the two AA whose synthesis is repressed by CcpA as deduced from the growth of the *ccpA* mutant. Taken together, in good agreement with the previous literature, our growth experiments underline the role of CodY as one of the most important nodes and the central regulator in the AA metabolism and SR of *S. aureus* (Table 2).

**Table 2:** Essential and modulated AA.

	AA esential in WT	AA essential in all Strains	AA synthesis inhibited in all Strains	AA synthesised in all Strains	AA synthesis uneffected in KO strains	AA synthesis inhibited by CodY	Essential AA due to CodY	AA synthesis inhibited by RSH	AA synthesis promoted by RSH	Compensated in quadruple mutant	Not compensated in quadruple mutant	RSH/codY effect (RSH–|GTP– > codY–|AA)	Additional inhibition by ccpA	AA synthesis dependent on ccpA
Ala-				X										
Arg-	X	X			X									
Asn-				X										
Asp-				X										
Cys-	X		X					X						
Gln-				X	X									
Glu-				X	X									
Gly-	X					X			X		X	X	X	X
His-									X	X				
Ile-				X					X	X				
Leu-	X					X	X							
Lys-				X	X									
Met-			X			X			X	X		X		
Phe-			X			X			X		X	X	X	X
Pro-	X	X			X									
Hydr.-Pro-/Pro-	X	X			X									
Ser-				X		X			X	X		X		
Thr-				X		X			X	X		X		
Trp-				X	X									
Tyr-						X			X	X		X		
Val-	X					X	X							

This table shows the essential AA as well as the influence of different central nodes on the synthesis of the different AA.

#### Adaptation to nutrient stress

**Figure 3:**
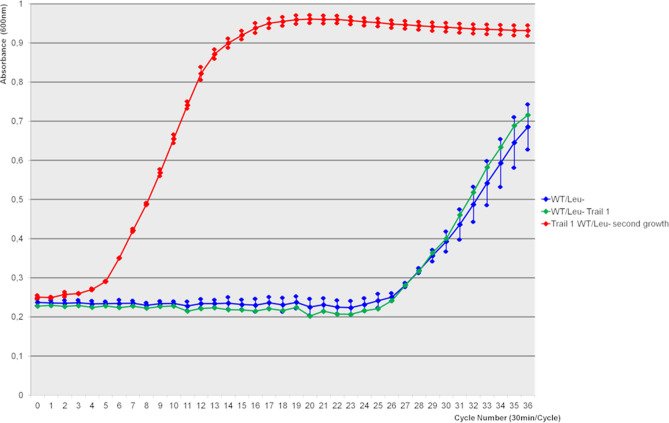
Secondary growth: The green curve represents the first growth trial of the WT under leucine deprivation. When growing the bacteria for the first time a strong increase of growth at the end of the experiment can be found. Blue shows the respective mean values. The red curve shows the growth of the same bacteria like in the green curve in a second growth experiment. Here the bacteria show a strong growth from the beginning suggesting that an adaptational process took place. In the supplemental material this effect is shown for different mutant strains in different AA deprived media.

For the *RSH* mutant under Glycine, Methionine, Valine and Leucine deprived conditions, for the WT and *ccpA* knockout strains when depriving them of Leucine and for the *ccpA*^***−***^ mutant deprived of Hydroxyproline as well as for the WT strain growing without Valine, growth resumed only at the very end of the incubation period. This late growth is very likely due to accumulation of compensatory mutations. When bacteria were re-grown in the corresponding AA limited media, no growth delay was observed (Fig. 3, Fig. S3).

### In silico network and dynamic simulations

#### A comprehensive network

The amino acid synthesis network was constructed by implementing all nodes, i.e. enzymes, AA and intermediate products known from biochemical^17^, protein interaction databases^18^ and literature to be important for SR and AA metabolism such as CodY, RSH, (p)ppGpp, CcpA, the amino acid biosynthetic operons (e.g. ilv operon and many more). Table S1 gives a detailed listing of all sources and literature references considered. The result is a network with 195 nodes and 320 edges representing a comprehensive overview of our recent knowledge about signalling cascades and the influence of different important nodes around the AA metabolism and the SR in *S. aureus.*

#### In silico identification of master regulators

Master regulators and thus the mutant strains used for these experiments were selected because of their proposed importance in the network. As in our previous studies^21^ network connectivity was used as a measure of influence on the network and thus as a predictor to identify these nodes.

**Figure 4:**
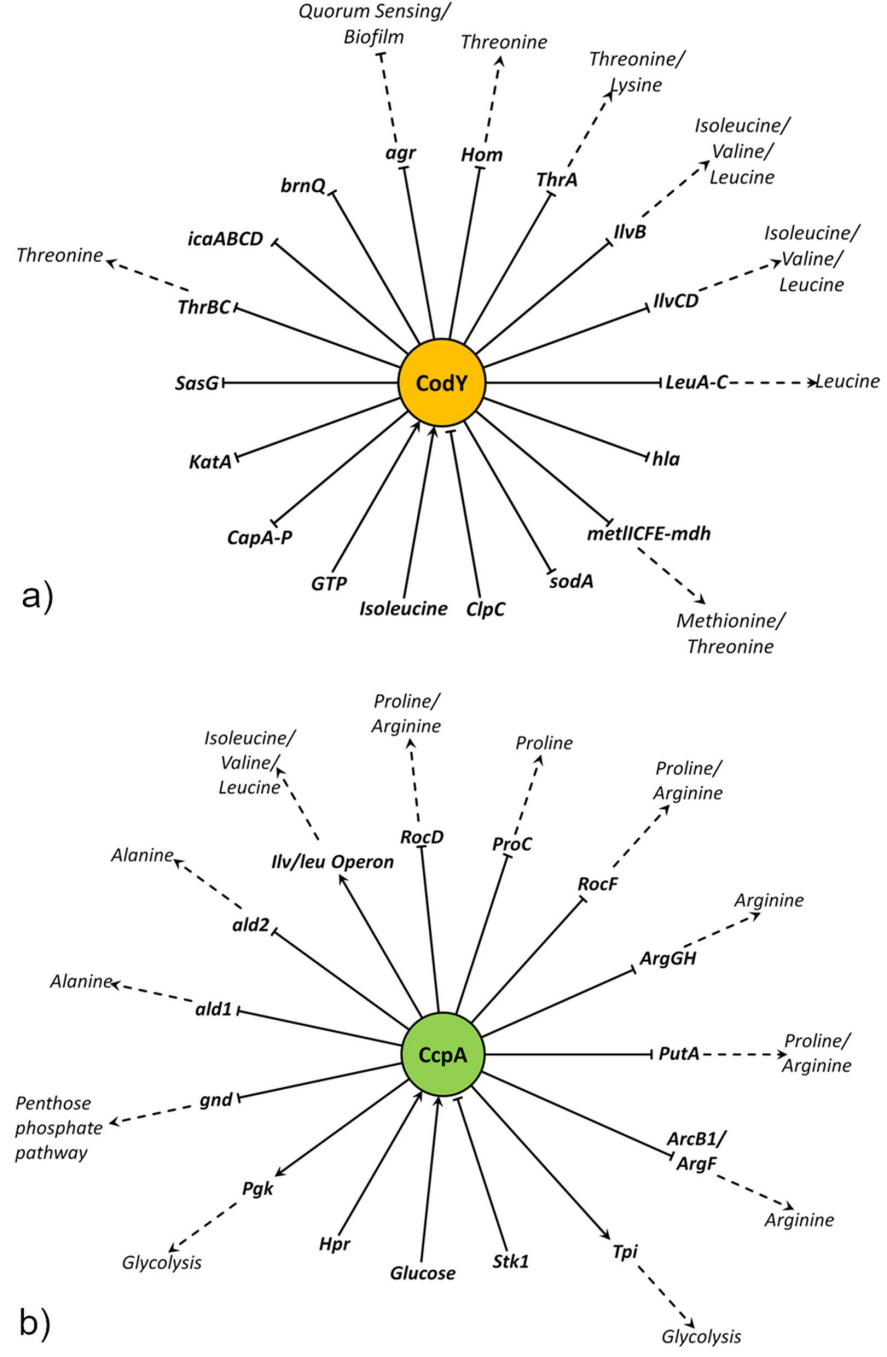
Efferent and afferent connections of CodY and CcpA : The efferent and afferent activating and inhibiting connections of CodY and CcpA are shown here as well as their influence on the AA-Synthesis.

Our model includes 15 operons controlled by CodY (15 efferent connections) and the effectors of CodY activity, GTP, Isoleucine and ClpC (3 afferent connections). CodY amongst numerous other genes, inhibits major AA biosynthesis gene clusters such as those involved Threonine, Methionine or branched-chain amino acid (BCAA) biosynthesis^5,13,15,27,28^. CodY repression can be relieved via RSH mediated drop of the GTP pool and/or via Isoleucine limitation^15^ (Fig. 4).

CcpA has 12 efferent and 3 afferent connections, is activated by Glucose and itself upregulates important enzymes within the glycolysis^29^. CcpA regulates via *gnd* the utilisation of glucose for the pentose phosphate pathway^29^. Moreover CcpA downregulates for example the alanine production^29,30^ and controls the transformation of proline into arginine and vice versa^10^. CcpA, besides regulating AA synthesis directly, also improves exploitation of glucose, producing metabolites important for the AA metabolism. Furthermore CcpA regulates the BCAA synthesis via the ilvDBC-leuABC-ilvA operon^31^ (Fig. 4).

The node with the third most interactions is (p)ppGpp. As for RSH enzymes of other bacteria, (p)ppGpp synthetase activity of RSH is likely induced by unloaded tRNA as consequence of AA limitation^32^. Besides de-repression of CodY target genes, (p)ppGpp inhibits translation^33^ and rRNA synthesis^34^ thus reducing resource consumption in cases of AA deprivation. The CodY-mediated effect of the SR is abrogated in a pppGpp0/CodY strain as it is not able to synthesise pppGpp.

Thus three master regulatory nodes were identified and successfully implemented in the network. These knockouts and a combination (*codY*, *ccpA*, *rsh*_*Syn*_ and pppGpp0/*codY*) were tested and their detailed behaviour was studied in silico in the simulation and also in in vitro in growth experiments.

### Combining and comparing in silico and in vitro results

#### Direct in silico–in vitro comparison

Growth of the knockout mutant strains in different AA deprived media was compared to the respective in silico AA production in an intermediate NW (in vitro to in silico semi quantitative consistency 62%). For a more realistic definitive network reflecting the real processes in *S. aureus* more genuine, modulatory input from further 12 interactions available from literature and including phylogenetic information on conserved modulatory interactions from related species had to be considered (details on the connectivity and references for these in Supplement, Table S1). In this network CcpA has an inhibiting connection to phenylalanine and glycine. CodY now inhibits additionally phenylalanine, tyrosine, THF and Serine. Cysteine is now activated by CodY. RSH now has suggested activating connections to phenylalanine, glycine and histidinol. Cysteine is inhibited by RSH. RelP/Q now inhibits histidine and methionine. Proline and arginine turned out to be clearly essential in the auxotrophic growth experiments. As the inability to produce the precursor ornithine turns both AAs to become essential as observed, we included this inactivation of the ornithine production into our simulation. These necessary changes in the simulation yield a much improved in vitro—in silico semi-quantitative consistency: A 81% agreement between AUC and the final network (NW) can be found (Table 3, Fig. 1).

**Table 3:**
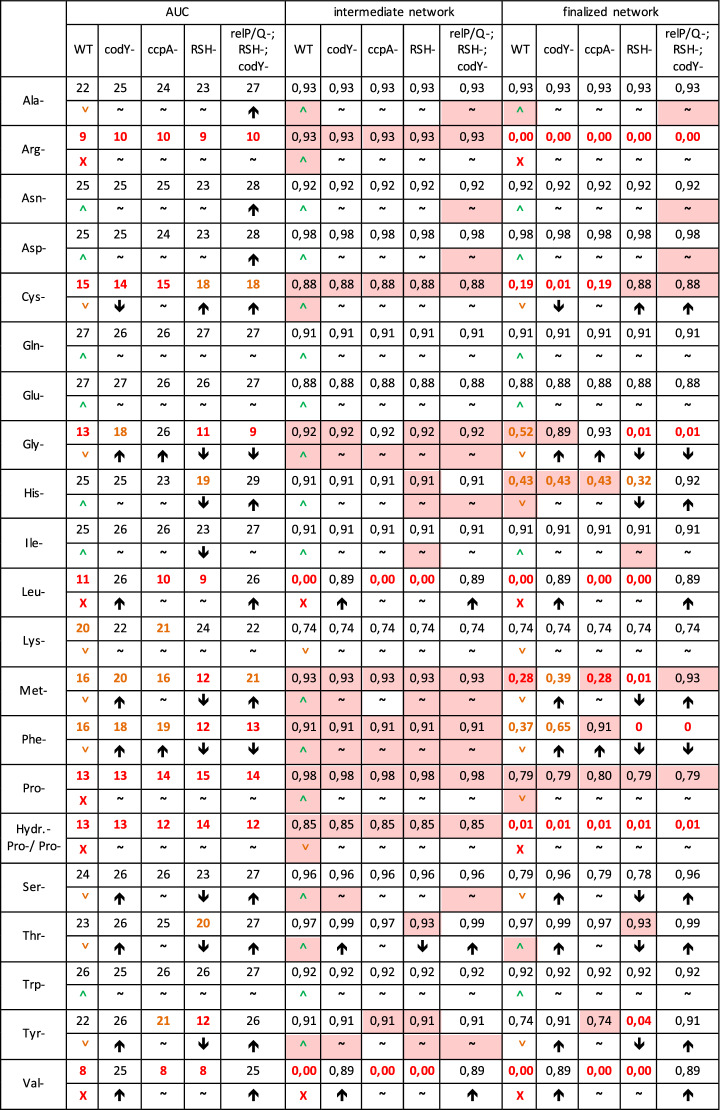
Semi quantitative and qualitative in vitro–in silico comparison.

The values were categorized for semi-quantitative evaluation via the AUC of the growth curves (essential AA: AUC 8–15 in red; average synthesis performance: AUC 16–21 orange; high synthesis performance: AUC 22–29 in black). The essential amino acids of the WT determined in this way are Arg, Cys, Gly, Leu, Pro, Val. Based on the AUC of the KO strains in the respective deficient media, the influence of the corresponding knocked-out node on the synthesis performance of the withdrawn AS can be evaluated. For the semi-quantitative evaluation of the in silico experiments the values were categorized too (essential AS 0.00–0.29 in red, average synthesis performance 0.30–0.69 in orange; high synthesis performance 0.7–1.0 in black). The semi-quantitatively mismatching results are highlighted in red. Moreover the qualitative evaluation of the growth curves of the WT is shown. With ˄, ˅ and X an uninhibited, an inhibited and no synthesis is indicated (each in comparison to the WT in full medium). For the KO strains, arrows indicate whether without this node the synthesis performance is impaired or improved or switched off compared to the WT. The in silico results (initial network and definitive network) were also assessed qualitatively in accordance with the qualitative assessment of the growth curves of the WT and the KO strains. The qualitatively inconsistent results are highlighted in red.

#### Evaluating remaining differences of this comparison

A categorization of results was done first. Categorization always results in an artificial fragmentation that may offer the potential for an incorrect scoring if not done carefully (see methods, considerations and procedures for the categorization; many other categorizations were evaluated within this process), Moreover, regarding our application, regulation of amino acid metabolism, we hence did focus on a primarily qualitative comparison. Finally, all raw data required for further such categorizations are listed in Table 3. In the additional Table S3 in the supplementary material, other categorization options and their semi-quantitative consistencies were compared in different examples.

After our categorization, there remained 19% in vitro—in silico discordances. These are further analyzed and investigated in Table S2: Only quantitative discordance (°) means that the simulation and the in vitro experiments qualitatively show the same results when evaluating the change in the respective AA synthesis strength induced by switching off the corresponding nodes. Categorization effect (*) means that small changes in the AA synthesis strength in vitro may eventually lead to categorization in a different growth strength category and thus to an artificially overestimated discordance found only in semi-quantitative yet not in qualitative comparisons. These two effects explain why all 15 semi-quantitative misfits no longer appear in the qualitative comparison. In this case the network must be more complex and detailed for a quantitative agreement. However, the data required for this do not currently exist (see discussion).

Overmodulation (˄) means that in vitro and in silico qualitatively the same effect is observed, yet the simulation shows activation quantitatively stronger than the in vitro growth results. Undermodulation (˅) however means that despite the qualitative concordance the simulation anyhow shows activation quantitatively weaker than the in vitro growth results.

In conclusion most misfits are found in the semi-quantitative comparison. But qualitatively in most of the cases a corresponding and matching effect can be observed when knocking out different network nodes.

#### Resulting high qualitative fit

A very good qualitative in vitro to in silico correspondence of the AA synthesis strength in the WT can be found in most cases. Similarly the change in synthesis performance by switching off the tested nodes is in silico almost always predicted correctly (Table 3, Fig. 1). The qualitative comparison yields a consistency of 92%. The simulation is a reliable and suitable simulation of SR and AA metabolism in *S. aureus*.

#### Seven qualitative growth curve patterns

Deprived of one single AA the strains show, qualitatively, seven different growth patterns suggesting seven patterns how the different central nodes influence the synthesis of these AA. In qualitative comparisons, these patterns can also be found correspondingly in silico (Table 3, Fig. 1). Growth without tyrosine is the prime example for the first pattern. Characteristic is a RSH as well as a CodY dependence. RSH inhibits the CodY expression which in turn upregulates the respective AA synthesis. This also means improved growth without CodY. Exemplary for the second pattern is the growth without leucine and valine which are only synthesized without CodY. Growth without essential AA like arginine represents the third pattern. The tested nodes have, if at all only little influence. The same applies to pattern four, which is characterized by strong growth of all strains and for which growth without asparagine is exemplary. Pattern five describes an activation, pattern six however an inhibition of the AA synthesis by RSH. Examples are growth without histidine and without cysteine respectively. Jet pattern six is not just the opposite of pattern five more the counterpart of pattern one. Pattern seven, for which growth without glycine is exemplary, resembles pattern one, however, with an additional inhibition of the respective AA synthesis by CcpA.

## Discussion

### Advantages and limitations of a Boolean network and a dynamic simulation

Due to the limited data available, a detailed simulation of the mechanisms, including, for example, enzyme kinetic data, is not possible. A Boolean network and, building on this, its dynamic simulation, even if maybe invalid in modeling cellular processes quantitatively, is an excellent option for at least a qualitative depiction of the real conditions when quantitative data are lacking. The analysis allows to reveal possible connections and making them understandable^35^. The Boolean network models the cellular decision processes but as on and off, yes or no, decisions. This is a clear limitation, as cellular processes happen with a whole range of dynamics between full activation and complete shutdown of activities, receptors or kinases. Hence, in a second step, dynamic simulations using Jimena interpolated between the on and off states using exponential functions to simulate the succession of events but also the intermediate states of activation for all proteins in the network and its logical connections. As detailed kinetics are only estimated by the simulation, these are semi-quantitative simulations, reliable on the implied order relations (e.g. what is first, later … and what is stronger, weaker…etc.) but allowing no accurate quantification. Hence, all our modeling is of a qualitative nature, which is clearly a limitation, but it works with the limited data which are currently available.

### Essential amino acids

#### Biological importance of AA metabolism

SR with its influence on the AA-metabolism allows *S. aureus* to adapt to a large variety of resources available in the vast variety of habitats in which it is able to grow^5,36^. The SR may also affect the biofilm building ability and virulence of *S. aureus* by activation of the quorum sensing system Agr, since Agr activity is modulated by CodY^13,21^. Yet, metabolism itself is important for virulence^37,39^. Thus SR and metabolism in general is an important factor making *S. aureus* and other bacteria so successful pathogens and causing so much medical problems. Due to the comprehensive representation of not only the SR but also the AA- and energy metabolism, the validated network presented here is a vantage point for further understanding and investigation of those complex processes.

#### AA essentiality

When an AA is found to be essential for growth, it indicates that the organism is not able to produce it on its own at least under the given growth conditions. Here we defined AA essentiality by lack of growth using a defined medium which is lacking single AAs. In previous studies different strains and media were used to determine which AAs are essential for *S. aureus* growth^40–44^. Arginine, Valine, Cysteine, Proline, Glycine and Leucine were frequently found to be essential which is well in accordance with our experiments and simulations (Table 4). Although Bois et al.^45^ describes the metabolism as highly conserved in the core genome, there are clear differences in the essentiality of the various AA in the 64 models he compared. Methionine, Cysteine, Proline and Asparagine in particular are often found to be essential. Less frequently, Histidine, Phenylalanine, Tyrosine, and Arginine are found to be essential, too. Thus just like our comparisons in Table 4 Bois et al.^45^ find a high degree of variability between the tested models and therefore with 64 models tested more essential AA in total. Glutamate and Valine are AA which have shown to be essential in our experiments and simulations, but not in that of Bois et al.^45^. On the other hand they found, at least under some circumstances, Methionine, Asparagine, Histidine, Phenylalanine, Tyrosine and Tryptophan to be essential which we haven’t found to be essential. With regard to Proline, Li et al.^11^ describe an auxotrophy in SA Newmann N315 although genes for Proline synthesis are present in the genome. Under certain circumstances, however, they also describe the occurrence of Proline prototrophic strains. Li et al.^11^ see an important role for this in regulatory mechanisms in which above all Ornithine and CcpA but also RocD, RocF and ProC are important. These interrelations are well reflected by our simulation and we also find a decisive role of these nodes, in particular of Ornithine, for the synthesis of Proline.

**Table 4:** Essential AA.

	Coutinho et al.^41^	Lincoln et al.^42^	Mah et al.^43^	Kurode et al.^40^	Kloos et al.^44^	Studies confirming essentiality	This work
ala				✓		1	
arg	✓	✓	✓	✓	✓	5	✓
cys	✓	✓	✓		✓	4	✓
gly		✓		✓		2	✓
his	✓					1	
ile				✓		1	
leu		✓			✓	2	✓
pro		✓	✓	✓	✓	4	✓
val	✓	✓	✓	✓	✓	5	✓

This table shows the essential AA for the WT in the in vitro experiments based on the AUC (AUC = 8–15; compare Table 3) and compares it to earlier findings.

#### Essential AA requirements resulting from SR regulation

The SR of *S. aureus* as well as its AA-metabolism have been well studied^5,16,46,47^. According to genome annotation and known operons, *S. aureus* has the genetic ability to produce all 20 proteinogenic AA on its own. Yet there are different strains in which different AA are described as essential in previous publications^40–43,48^. In our new study we can show that the observed essentially is in large part due to repression via CodY. AA essentiality is thus based on regulatory effects. The network presented here reflects these well and makes the complex regulation comprehensible in terms of logical switches, connected pathway and key regulatory genes. This includes the inhibition of valine or leucine synthesis by CodY or the activation of histidine synthesis by RSH. Thus, many of the observable results in AA synthesis are indeed not due to defects in the synthesis pathway, but rather to emergent effects through the interaction of the various nodes. This explains the high diversity between the different strains and underlines the adaptability of *S. aureus* to different environmental conditions. As shown in our results the *S. aureus* strain investigated here is able to switch from essentiality to sufficient synthesis in less than 24 h. Thus, although *S.aureus* has the genetic equipment to synthesize all the proteinogenic AA some responsible genes are only insufficiently expressed. It is precisely these complex relationships that cannot be found in the individual consideration of interactions. Hence in the next step experimental in vitro results were compared to in silico simulation results to see to what extent these emergent effects can be explained and investigated by the network. Due to the comprehensive representation of all relevant pathways and their interdependencies the simulation can thus give a better insight into the regulation of these metabolic processes.

#### Adaptation to nutrient stress

In some of the trials we observed growth at the very end of the experiment. When regrowing these bacteria after sub-culturing we saw that these bacteria were able to immediately grow in the AA limited condition with exponential growth soon after the beginning, indicating a process through which the bacteria adapted to the new environmental conditions in less than 24 h. Coutinho et al.^41^ also observed a almost 24 h delay in exponential growth and called these AA "semi essential" yet without giving a mechanistic explanation.

### Detailed network analysis: in silico optimal and less well represented in vitro results

In the following, we discuss in detail remaining challenges of the refined SR network model when comparing it qualitatively to the in vitro results.

#### Challenges of specific AA synthesis pathways

Cases with a semi-quantitative misfit that cannot be found in a qualitative comparison most likely result from a quantitatively imprecise simulation, yet in which the connectivity of the nodes can be assumed to be correct. Qualitatively the in vitro AA synthesis of the WT does not match the in silico results in alanine, histidine, threonine and proline. For the first three of them however a relevant synthesis strength is predicted correctly by the simulation. Taking the WT in full medium as reference one would expect the histidine synthesis to be predicted in silico marginally higher. The simulated synthesis of alanine and threonine on the other hand is in silico marginally higher predicted than it turns out to be in the in vitro experiments. The in silico proline synthesis is predicted to be too effective. The importance of proline synthesis from hydroxyproline seems to be overestimated in silico because the prediction is appropriate if in addition to proline also hydroxyproline is switched off.

#### Challenges of central regulators effects

In three out of four cases (alanine, asparagine and aspartate) in which switching off the nodes in silico does not qualitatively predict the effect as it can be observed in vitro it is the effect of the quadruple mutant and thus the knocking out of four nodes (relP/Q-; RSH-;codY-) at the same time. The qualitative effects of turning off each node individually are simulated correctly. Thus, the mismatch in the synthesis strength of the quadruple mutant most likely results from systemic effects, e.g., the incorrect respective weighting of the correctly represented interactions of these nodes against each other. Yet in the fourth case isoleucine has a slightly reduced synthesis performance, when knocking out RSH which is in silico qualitatively not reproduced. In all these cases both inappropriate weighting of the connections in the pathway yet also a missing node could account for that. Further in detail investigation of these pathways is needed in future, using the simulation for hypothesis generation as well as fast and easy testing of different hypotheses.

#### Challenges of simulation improvement

For an even more precise simulation, firstly not yet available kinetic data is needed to weight the interactions. This applies in particular, if we would like to parametrize the e-functions we use in the simulation directly or even use other, more complex functions for the model. Second, it must be considered that the current state of knowledge regarding the interactions of the nodes is probably not yet comprehensive and third, inputs from outside like sensing environmental signals^49^ need to be considered, which could not be incorporated due to the limited network size. In the future new connections which will further improve the predictive power of the simulation must be expected to be found, possibly even through a targeted search based on this simulation. Yet despite the understandable aim for improving the predictive power of the simulation it always has to be considered, that it must remain a simplified simulation and representation of reality in order to do its job which is, for example, the development and preliminary testing of hypotheses. However, whether the simulation itself gets better is always a matter of whether the increasing complexity also leads to a relevant improvement in the predictive power. With a qualitative agreement of 92% using simple interactions without kinetic properties, this simulation has the optimal trade off in this regard. An additional insertion of kinetic data and thus an extreme increase in the complexity of the simulation has only a very low potential for improvement (8%). Thus, when weighing complexity and goodness of fit, a balance that can hardly be improved has been reached. As a consequence, the exponential functions of this method once again prove to sufficiently reflect reality, like already shown in numerous previous studies (e.g. Czakai et al.^25^).

The same applies to the simulation margins, no simulation can reflect the entire reality but is naturally limited. Of course, there will always be inaccuracies at these margins. Enlargement of the network only shifts these inaccuracies at the price of increasing complexity. This simulation with its 195 nodes and 320 edges for the representation of such complex mechanisms hence offers a good tradeoff between accuracy and model reduction.

### Key regulators

The network focuses on key regulatory nodes with great impact on nutrient stress response. This includes nodes important for the SR but also for the AA—and the energy metabolism like CodY, RSH and Ccpa but also the Met- and the Ilv-Operon and many more. These in the literature stronger represented nodes are much better examined and discussed than rather peripheral nodes and therefore have a more precise representation in the network. The distance to these central nodes moreover increases the probability that edges and possibly also nodes have not been discovered and described so far and thus are missing in the network. However a lot of detail is provided by the model, for example when looking at all the peripheral interactions and nodes included. Another example is the in detail represented glycolysis pathway providing important intermediates for the AA biosynthesis. Nevertheless, this model still remains simplified. It obviously does not represent the exact interaction mechanisms on a molecular level yet just the effect one node has on another node (activating/inhibiting) is considered. As a result of this the model works without detailed kinetic data which are often not available and experimentally hard to obtain. However, this model not only provides a platform for simulation of SR and AA-Metabolism in *S. aureus* it also helps to better elucidate in combination with published data and our own experiments the function and interactions of the central regulatory nodes.

### Implications for *S.aureus* antibiotic treatment

Our model supports novel antibiotic targeting strategies either against SR or the AA metabolism and the modeled further network components. This helps to fight resistance in *S. aureus* infection, and, by virtue of the high conservation of AA metabolism, against Gram positive bacteria in general.

There are already antibiotics like Cotrimoxazole^50^ acting directly on the AA-Metabolism and the SR of *S. aureus*. Knowing the mechanisms behind the *S. aureus* stress response and being able to simulate it allows identifying all essential points in the network, depending on the exact strain and the environmental conditions and disrupting the connected AA pathway only in *S. aureus*. However, more efficient strategies considering SR and regulation in general are possible. When combining our recent model with our models on Quorum-Sensing (QS) and biofilm formation in *S. aureus*^21^ it is obvious that the nutrient stress in *S. aureus* has also a strong impact on QS and biofilm formation and thus on the virulence of *S. aureus* in general. Nutrient stress is shown to lead to de-repression of CodY target genes^13^ including Agr activity. Thus, there is a direct link between central metabolism and virulence gene expression and biofilm formation^21^. Thus nutrient stress reduces biofilm formation turning *S. aureus* towards the easier to treat planktonic state. Hence, a second new antibiotic strategy implied by our modeling would be to use nutrient stress or targeting the SR regulatory proteins directly by new compounds^51^. Such sensitizers to make *S. aureus* more vulnerable and less virulent would be novel and could again probably be used against other bacteria. Dysregulation or hyper activation of bacterial proteins with damaging consequences is in general a promising strategy^52^. A third strategy combines our model with our studies on toxic metabolic intermediates^53^ and targets our network such that toxic intermediates of the AA metabolism accumulate. This can either be achieved by activating the enzymes of the AA metabolism which have a low Michaelis–Menten constant and produce the more toxic intermediates or inhibiting the directly following AA metabolizing enzymes so that the toxic intermediate accumulates. All these different strategies can now be modeled and studied in silico with our model, even transferred to other infections such as fungal infections or gram negative bacteria. Moreover, the most promising compounds can be synthesized and used as leads for novel antibiotics in particular against Gram positive bacteria, and, most importantly, MRSA. With a more selective therapy well known side effects of an aggressive antibiotic therapy like e.g. pseudomembranous colitis, an infection of the colon due to antibiotic induced imbalance in the bacterial colon flora, may be avoided^54^.

## Conclusion

Here we present a model of SR. We analyze amino acid metabolism (AA) and key regulators such as CodY and CcpA, as well as the Ilv- and the Met-operon and their effects. The network model and its dynamics agree well with data from literature and databases. This is validated directly by conducting growth curve experiments with different AA metabolism mutants (quantitative agreement 81%, qualitative 92% of all tested conditions). The network is fully made available, is easily simulated and presents a good and reliable representation of SR and regulated processes in AA and energy metabolism in *S. aureus* including testing and modeling the effect of various mutations. The AA essentiality in the WT and in the mutant strains is delineated in the simulation and well supported by the experimental data. Our data support a fast adaptation mechanism by which *S. aureus* can adapt its AA metabolism in less than 24 h. The in silico model allows also to develop new antibiotic targeting strategies on AA metabolism including its regulation or accumulation of toxic intermediates for *S. aureus* and MRSA.

## Supplementary Information

Supplementary Information 1.

Supplementary Information 2.

## Data Availability

All data analyzed in this paper are contained in the manuscript and its supplementary files. They are fully available to the public.
