# Supplementary material for: Modeling of stringent-response reflects nutrient stress induced growth impairment and essential amino acids in different Staphylococcus aureus mutants
Source: Sci Rep. 2021 May 6;11:9651. doi: 10.1038/s41598-021-88646-1 (PMC8102509; doi:10.1038/s41598-021-88646-1)
Supplement: Supplementary file 1 — Supplementary Information 1. [file 41598_2021_88646_MOESM1_ESM.docx]

**Modeling of Stringent-Response reflects nutrient stress induced growth impairment and essential amino acids in different *Staphylococcus aureus* mutants**

Christof Audretsch^1,2^, Fabio Gratani^1^, Christiane Wolz^1,^* and Thomas Dandekar^2,3,^*

^1^Institut für Med. Mikrobiologie und Hygiene; Elfriede-Aulhorn-Straße 6; 72076 Tübingen

^2^Department of Bioinformatics, Biocenter, Am Hubland, University of Würzburg, 97074 Wuerzburg, Germany.

^3^EMBL Heidelberg, Bioinformatics, Meyerhofstraße 1, 69117 Heidelberg, Germany

***Correspondence to:** dandekar@biozentrum.uni-wuerzburg.de; Fax: +49 931 318 4552;
Tel: +49 931 318 4551; christiane.wolz@med.uni-tuebingen.de Tel: 49 7071 2974648**;**

**Supplementary material**

**Independent offered Files:**

**Online E-Supplement Figure 1E: Scalable high-resolution figure of the network**

This supplement shows the Network as we constructed it from knowledge of different publications. Red nodes are AAs, green nodes are intermediate metabolites. Blue boxes represent the and-gates. The suggested modifications are shown in pink.

**Files contained in this supplementary document:**

**Table S1: Nodes and edges**

This file gives references for all activating or inhibiting connections and nodes modelled in the network. The first column shows all the nodes of the simulated network. In the second column the activating efferences and in the third column the inhibiting efferences with their respective references are shown.

**Table S2: Discordant Results**

This file evaluates and explains why 19% of the cases are not yet appropriately modelled in the network. Thus it shows which discordant results remain in the current model and gives clear reasons for this discordance. Moreover it proposes further possible changes potentially leading towards a perfect fit of the network, yet within this table also problems emerging from these changes (most often incomplete data or inconsistencies) are noted leading us to reject these proposals.

**Table S3: Testing different parameters for consistency of simulation with experimental results**

Here the consistencies with different numbers of categories and thresholds are shown. For our studies we used three categories and the corresponding thresholds, because categorization in weak, medium and high synthesis feels intuitive and still provides the necessary information content, yet without losing the semi-quantitative character, which is a risk when the number of categories increases.

**Figure S1 a-u: In vitro and in silico comparison**

In the upper part of each figure the data of the growth curve experiment with the medium lacking the respective AA is shown. Here the different knock out mutants (WT; CcpA-, rsh-; CodY-; relP/Q-,CodY-, rsh-,) are indicated by the different coloured curves. In the middle and the lower part of each figure the in silico synthesis of the respective AA is shown. The graphic in the middle shows the data of the first network and the lower graphic shows the data of the definitive network (Jimena version from 26.02.2015; <https://www.biozentrum.uni-wuerzburg.de/bioinfo/computing/jimena/>) [61]


. The graphic can be zoomed to display the figure and the legend in detail. The in vitro time steps are each 30 minutes (p.12 Ala-, p.13 Arg-, p.14 Asn-, p.15 Asp-, 16 Cys-, p.17 Gln-, p.18 Glu-, p.19 Gly-, p.20 His-, p.21 Ile-, p.22 Leu-, p.23 Lys-, p.24 Met-, p.25 Phe-, p.26 Pro-, p.27 Hydroxy Pro-/Pro-, p.28 Ser-, p.29 Thr-, p.30 Trp-, p.31 Tyr-, p.32 Val-)

**Figure S2 a-e: Growth curves**

Growth curves of the different mutant strains (a: WT (p.31); b: CodY- (p.32);c: CcpA- (p.33); d: rsh- (p.34); e: relP/Q-, CodY- rsh- (p.35)) for individual experiments are shown. The missing AA from the medium is indicated. The graphic can be zoomed to display the figure and the legend in detail. The time steps are each 30 minutes.

**Figure S3: Secondary growth**

In green the growth curve of different mutant strains in different AA deprived media are shown. All of them show a strong increase of growth at the end of the experiment. Blue shows the respective mean values. The red curve shows the growth of the same Bacteria like in the green curve in a second growth experiment. Here the bacteria show a strong growth from the beginning suggesting that an adaptational process took place.

**Figure S4: Boolean AND logic gate**

Here the AND logic gate is shown; including the respective truth table, the in silico construction, the Boolean annotation and pictogram as well as the reaction of the nodes in the simulation. Below the simulation graph the respective perturbations are shown (Jimena version 26.02.2015; <https://www.biozentrum.uni-wuerzburg.de/bioinfo/computing/jimena/>) [61].

**Table S1:**

| **Node** | **activating efference to:** | **inhibiting efference to:** |
| --- | --- | --- |
| (p)ppGpp | psmα1-4[8] psmβ1,2[8] | tsf[6] rpsL[6] rpsB[8] infB[6] guaC[6] DnaG[9] |
| 2-Oxoglutarate | Glutamate[18,5,30,31] |  |
| 3-Phosphoglycerate | Phosphoenolpyruvate[18,5] Serine[32,33] |  |
| 6-P-Gluconate | Ribulose-5-Phosphate[34] |  |
| 6-P-Gluconolactone | 6-P-Gluconate[35,36] |  |
| ABC transporter | - | - |
| acetyl-CoA | Citrate[18,5,30] |  |
| AcnA | 2-Oxoglutarate[18,30] |  |
| agr | - | - |
| AHB | Isoleucine[32,37,7,4] |  |
| Alanine | AS-loaded tRNAs[38] Pyruvate[18] | tRNA[38] |
| ald1 | Alanine[18] Pyruvate[18] |  |
| ald2 | Alanine[18] Pyruvate[18] |  |
| alpha-Ketobutyrate | AHB[32,37,7,4] |  |
| alpha-Ketoglutarate | - | - |
| alpha-Ketoisovalerate | Valine[32,37,7,4] Leucine[32,7,4] |  |
| ArcB1/ArgF | Citrulline[11,32] |  |
| ArgGH | Arginine[11,32] |  |
| Arginine | Ornithine[11] AS-loaded tRNAs [38] | tRNA[38] |
| ArgJBCD | Ornithine[11,32] |  |
| AroACL | Chorismate[32,39] |  |
| AroBDEF | Shikimate[32,39] |  |
| ASAT | Aspartate[40,41] |  |
| Asd | Aspartate Semialdehyde[32,42] |  |
| AS-loaded tRNAs | translation[38] |  |
| asnA | Aspartate[40,41] |  |
| Asparagine-tRNA-Asn | Translation[38] Aspartate[40,41] |  |
| Aspartate | Aspartate Semialdehyde[32,42] Aspartate-tRNA-Asn[43,44] |  |
| Aspartate Semialdehyde | Lysine[32,42] Homoserine[32,42] |  |
| Aspartate-tRNA-Asn | Asparagine-tRNA-Asn[43,44] Translation[38] |  |
| Asp-tRNA-Synthetase | Aspartate-tRNA-Asn[43,44] |  |
| ATP | c-di-AMP[45] |  |
| brnQ1 | - | - |
| capA-P | - | - |
| CcpA | Tpi[5] Pgk[5] ilvDBC-leuABC-ilvA[17] | ArgGH[11] RocD[11] RocF[11]] ProC[11] PutA[11] arcB1/ArgF[11] gnd[5] ald1[5] ald2[5] |
| c-di-AMP | rsh[46] |  |
| Chorismate | Indolglycerol-P[32,39,47] Tyrosine[32,39,48] Phenylalanine[32,39,48] |  |
| Citrate | 2-Oxoglutarate[18,30] |  |
| Citrulline | Arginine[11,32] |  |
| clpC |  | CodY[49] |
| CodY |  | ilvCD[6] ilvB[6] ThrBC[14] hla[15] icaABCD[15] brnQ1[6]  sodA[4] capA-P[4] KatA[4]] sasG[4] LeuA-C[6] metICFE-mdh[16] agr[15] ThrA[4] Hom[4] |
| collagen | Proline[11] |  |
| CysEKM | Cysteine[32,50] |  |
| Cystathionine | Homocysteine[32,51,52] Pyruvate[32,51,52] |  |
| Cysteine | Cystathionine[32,51,52] AS-loaded tRNAs[38] | tRNA[38] |
| DacA | c-di-AMP[45] |  |
| DapABCDEX | Lysine[32,42] |  |
| Dat | Alanine[18,53] Pyruvate[18,53] Glutamate[18,5,30,31] |  |
| DHAP | GA3P[18,5,30] |  |
| Diaminopyrimidine |  | folA[27] |
| DnaG | Replication[9] |  |
| drp35 | 6-P-Gluconate[35,36] |  |
| Eno | Phosphoenolpyruvate[18,5] |  |
| Erythrose-4-Phosphate | Sedoheptulose-7-Phosphate[35,54] GA3P[35,54] Shikimate[32,39] |  |
| FbaA | DHAP[18,5,30] GA3P[18,5,30] |  |
| FemC | Glutamine[18] |  |
| folA | THF[27] |  |
| folP | THF[27] |  |
| Fructose-6-Phosphate | DHAP[18,5,30] GA3P[18,5,30] Xylose-5-Phosphate[34] Erythrose-4-Phosphate[34] Sedoheptulose-7-Phosphate[35,54] |  |
| GA3P | 3-Phosphoglycerate[18,5,30] Xylose-5-Phosphate[34] Erythrose-4-Phosphate[34] Ribose-5-Phosphat[34] |  |
| GapA1 | 3-Phosphoglycerate[18,5,30] |  |
| GatCAB | Asparagine-tRNA-Asn[43,44] Glutamine-tRNA-Gln[43,44] |  |
| GdpP |  | c-di-AMP[45] |
| Glk | Glucose-6-Phosphate[18,5,30] |  |
| Gln-tRNA-Synthetase | Glutamine-tRNA-Gln[43,44] |  |
| GltA | Citrate[18,5,30] |  |
| Glucose | CcpA[5] Glucos-6-Phosphate[18,5,30] |  |
| Glucose-6-Phosphate | 6-P-Gluconolactone[34,55] Fructose-6-6Phosphate[18,5,30] |  |
| Glutamate | Ornithine[11,32] Glutamine[18,32] Glutamate-tRNA-Gln[43,44] alpha-Ketoglutarate[32,56] Histidinol-P[32,56] |  |
| Glutamate-tRNA-Gln | Glutamine-tRNA-Gln[43,44] Translation[38] |  |
| Glutamine | Glutamine-tRNA-Gln[43,44] |  |
| Glutamine-tRNA-Gln | Translation[38] |  |
| Glu-tRNA-Synthetase | Glutamate-tRNA-Gln[43,44] |  |
| GlyA | Serine[18,32] THF[18,32] Glyceine[18,32] |  |
| Glycine | Serine[18,32] N5-N10-Met-THF[18,32] AS-loaded tRNAs[38] THF[18,32] | tRNA[38] |
| gnd | Ribulose-5-Phosphate[34] |  |
| GTP | CodY[7] |  |
| guaC | - | - |
| HisAEGHI | IG3P[32,41] |  |
| HisB | Imidazolacetal-P[32,56] Histidinol[32,56] |  |
| HisC | alpha-Ketoglutarate[32,56] Histidinol-P[32,56] |  |
| HisD | Histidine[32,41] |  |
| Histidine | AS-loaded tRNAs[38] | tRNA[38] |
| Histidinol | Histidine[32,41] |  |
| Histidinol-P | Histidinol[32,56] |  |
| hla | - | - |
| Hom | Homoserine[42,51,16] |  |
| Homocysteine | Methionine[32,51,52] |  |
| Homoserine | Threonine[18,32] Cystathionine[51,52] |  |
| Hpr | CcpA[57] |  |
| icaABCD | PIA[58] |  |
| Icd | 2-Oxoglutarate[18,30] |  |
| IG3P | Imidazolacetal-P[32,56] |  |
| ile-tRNA | Translation[38] | ABC transporter[7] |
| Ile-tRNA-Synthetase | ile-tRNA[6] | tRNA[6] |
| IlvA | Pyruvate[18] alpha-Ketobutyrate[32,7,4] |  |
| IlvB | AHB[32,37,7,4] alpha-Ketoisovalerate[32,37,7,4] |  |
| IlvCD | Isoleucine[32,37,7,4] alpha-Ketoisovalerate[32,37,7,4] |  |
| ilvDBC-leuABC-ilvA | LeuA-C[14] ILVCD[14] IlvB[14] |  |
| IlvE | Isoleucine[32,37,7,4] Valine[32,37,7,4] Leucine[32,7,4] |  |
| Imidazolacetal-P | alpha-Ketoglutarate[32,56] Histidinol-P[32,56] |  |
| Indolglycerol-P | Tryptophane[32,39,47] GA3P[32,39,47] |  |
| Indolmycin |  | trp-tRNA Synthetase[3] |
| infB | Translation[6] |  |
| Isoleucine | ile-tRNA[6] CodY[4] | tRNA[6] |
| KatA | - | - |
| LeuA-C | Leucine[32,7,4] |  |
| Leucine | AS-loaded tRNAs[38] | tRNA[38] |
| LysA | Lysine[32,42] |  |
| LysC | Aspartate Semialdehyde[32,42] |  |
| Lysine | AS-loaded tRNAs[38] | tRNA[38] |
| MetC | Homocysteine[32,51,52] Pyruvate[32,51,52], |  |
| MetEH | Methionine[32,51,52] |  |
| MetF | N5-Met-THF[18,32,52] |  |
| Methionine | met-tRNA[16,16] SAM[32,51,52] | tRNA[16] |
| metICFE-mdh | MetL[16] MetX,I[16] MetC[16] MetEH[16] MetK[16] MetF[16] |  |
| MetK | SAM[32,51,52] |  |
| MetL | Aspartate Semialdehyde[32,42] Homoserine[32,42] |  |
| met-tRNA | Translation[38] |  |
| Met-tRNA-Synthetase | met-tRNA[16] | tRNA[16] |
| MetX,I | Cystathionine[51,52] |  |
| Mupirocine |  | Ile-tRNA Synthetase[3] |
| N5-Met-THF | Methionine[32,52] |  |
| N5-N10-Met-THF | THF[18,32] Serine[18,32] N5-Met-THF[18,32] |  |
| Ornithine | Citrulline[11,32] Proline[11,32] |  |
| Oxalacetate | Citrate[18,5,30] Aspartate[40,41] |  |
| PABA | THF[27] |  |
| PdhABCD | acetylCoA[18,5,30] |  |
| PfkA | DHAP[5,30] GA3P[5,30] |  |
| Pgi | Fructose-6-Phosphate[18,5,30] |  |
| Pgk | 3-Phosphoglycerat[18,5,30] |  |
| Pgm | Phosphoenolpyruvate[18,5] |  |
| PheA | Phenylalanine[32,39] |  |
| Phenylalanine | AS-loaded tRNAs[38] | tRNA[38] |
| Phosphoenolpyruvate | Pyruvate[18,5] Shikimate[32,39] Chorismate[32,39] |  |
| PIA | - | - |
| ProC | Proline[11] |  |
| Proline | AS-loaded tRNAs[38] Ornithine[11] | tRNA[38] |
| PRPP | Indolglycerol-P[32,39,47] IG3P[32,41] |  |
| prsA | PRPP[34] |  |
| psmALPHA1-4 | - | - |
| psmBETA1,2 | - | - |
| PutA | Ornithine[11] |  |
| Pyc | Oxalacetate[18,5,30] |  |
| Pyk | Pyruvate[18,5] |  |
| Pyruvate | Alanine[18] AHB[32,37,7,4] acetylCoA[18,5,30] Oxalacetate[18,5,30] alpha-Ketoisovalerate[32,37,7,4] |  |
| relP/Q | (p)ppGpp[10] | GTP[10] |
| Replication | - | - |
| Ribose-5-Phosphate | PRPP[34] |  |
| Ribosome | Translation[38] |  |
| Ribulose-5-Phosphate | Ribose-5-Phosphate[55] |  |
| RocD | Proline[11] Ornithine[11] |  |
| RocF | Ornithine[11] |  |
| rpe | Ribulose-5-Phosphate[59] |  |
| rpiA | Ribose-5-Phosphate[55] |  |
| rpsB | Ribosome[8] |  |
| rpsL | Ribosome[6] |  |
| rsh | (p)ppGpp[6] | GTP[6] |
| SAM | - | - |
| sasG | - | - |
| ScpA | Proline[11] |  |
| Sedoheptulose-7-Phosphate | Ribose-5-Phosphate[34] Xylose-5-Phosphate[34] |  |
| SerABC | Serine[32,33] |  |
| Serine | Cysteine[32,50] Glycinee[18,32] Pyruvate[18] Tryptophane[32,39,47] GA3P[32,39,47]  AS-loaded tRNAs[38] | tRNA[38] |
| Shikimate | Chorismate[32,39] |  |
| sodA | - | - |
| SspB | Proline[11] |  |
| Stk1 |  | CcpA[57] |
| Sulfonamide |  | folP[27] |
| tal | Sedoheptulose-7-Phosphate[35,54] GA3P[35,54] |  |
| THF | Glycine[18,32,52] |  |
| ThrA | Aspartate Semialdehyde[32,42] Homoserine[32,42] |  |
| ThrBC | Threonine[18,32,4] |  |
| Threonine | AS-loaded tRNAs[38] alpha-Ketobutyrate[32,7,4] | tRNA[38] |
| tkt | Xylose-5-Phosphate[34] Erythrose-4-Phosphate[34] Ribose-5-Phosphate[34] |  |
| Tpi | GA3P[18,5,30] |  |
| Translation | - | - |
| tRNA | rsh[8,6,3] |  |
| tRNA Synthetase | AS-loaded tRNAs[38] | tRNA[38] |
| tRNA-Asn | Aspartate-tRNA-Asn[43,44] |  |
| tRNA-Gln | Glutamate-tRNA-Gln[43,44] |  |
| TrpAB | Tryptophane[32,39,47] GA3P[32,39,47] |  |
| TrpCDEF | Indolglycerol-P[32,39,47] |  |
| trp-tRNA | Translation[38] |  |
| Trp-tRNA-Synthetase | trp-tRNA[3] | tRNA[3] |
| Tryptophane | trp-tRNA[3] | tRNA[3] |
| tsf | Translation[38] |  |
| TyrA | Tyrosine[32,39] |  |
| TyrB | Tyrosine[32,48] |  |
| Tyrosine | AS-loaded tRNAs[38] | tRNA[38] |
| Valine | AS-loaded tRNAs[38] | tRNA[38] |
| VitB12 | Methionine[32,52] |  |
| Xylose-5-Phosphate | Ribulose-5-Phosphate[59] |  |
| YbbR | c-di-AMP[60] |  |
| zwf | 6-P-Gluconolactone[34,55] |  |

**Table S2:**

| **AA lacking** | **mutant strain** | **reason for discordance** | **kind of discordance** | **possible solution** | **why not implemented** |
| --- | --- | --- | --- | --- | --- |
| cys | rsh- | overmodulated˄ | quantitative discordance° | change influence strength | no kinetic data |
|  | RelP,Q-; rsh-; Cody- |  |  |  |  |
| gly | WT | overmodulated˄ | quantitative discordance° | change influence strength | no kinetic data |
|  | CodY- |  |  |  |  |
| his | WT | undermodulated˅ | quantitative discordance° | change influence strength | no kinetic data |
|  | CodY- |  |  |  |  |
|  | CcpA- |  | categorization effect* | improve categories | misfit elsewhere |
| lys | WT | undermodulated˅ | categorisation effect* | improve categories | misfit elsewhere |
|  | CcpA- |  |  |  |  |
| met | WT | undermodulated˅ | quantitative discordance° | change influence strength | no kinetic data |
|  | CcpA- |  |  |  |  |
|  | RelP,Q-; rsh-; Cody- | overmodulated˄ |  |  |  |
| phe | CcpA- | overmodulated˄ | quantitative discordance° | change influence strength | no kinetic data |
| thr | rsh- | overmodulated˄ | quantitative discordance° | change influence strength | no kinetic data |
| tyr | CcpA- | overmodulated˄ | categorization effect* | improve categories | misfit elsewhere |

**Table S3. Testing different parameters for consistency of simulation with experimental results**

| Number of categories | Threshold parameters | Semi-quantitative consistency [%] |
| --- | --- | --- |
| II | weak synthesis: in silico 0,00-0,49  / AUC 8-18;  high synthesis: in silico 0,50-1,00  / AUC 19-29 | 86 |
| III | weak synthesis: *in silico* 0,00-0,29  / AUC 8-15;  medium synthesis: *in silico* 0,30-0,69  /AUC 16-21;  high synthesis: *in silico* 0,7-1,0  / AUC 22-29 | 81 |
| IV | weak synthesis: *in silico* 0,00-0,24  / AUC 8-13;  moderate synthesis: *in silico* 0,25-0,49 /AUC 14-18;  elevated synthesis: in silico 0,50-0,74  /AUC 19-23;  high synthesis: *in silico* 0,75-1,0  / AUC 24-29 | 71 |


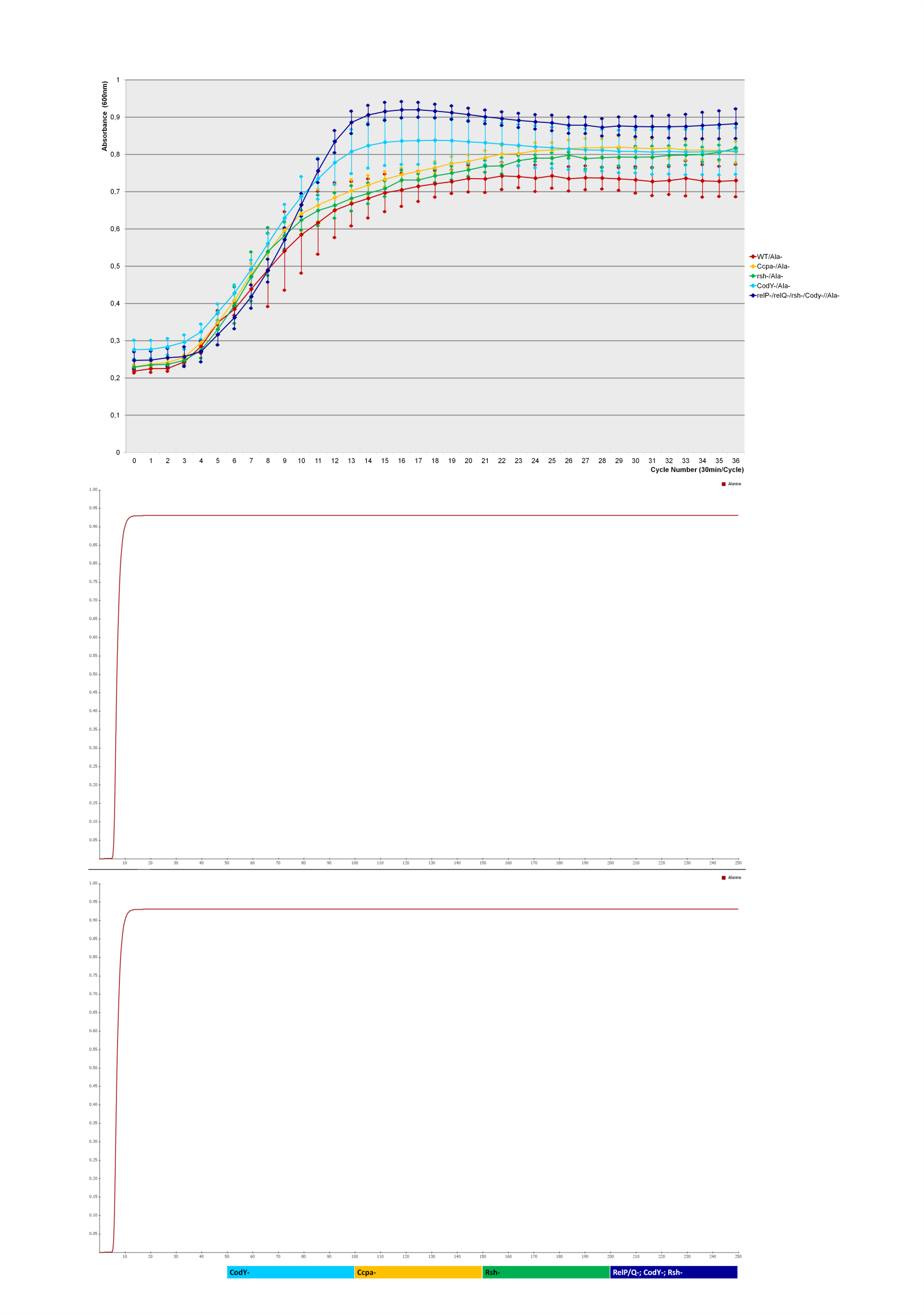


**Figure S1 a-u:**

S1a) Ala-


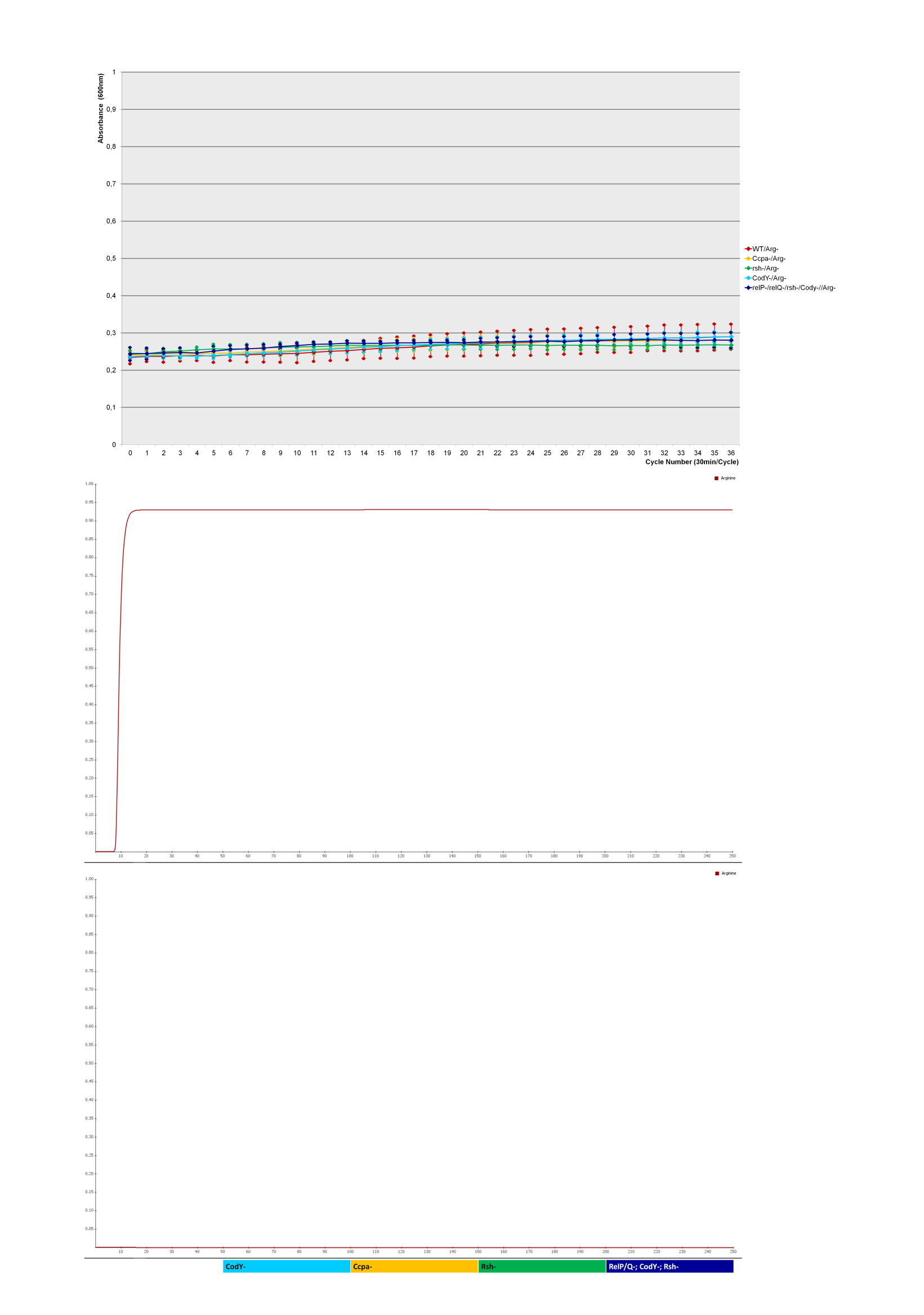


S1b) Arg-


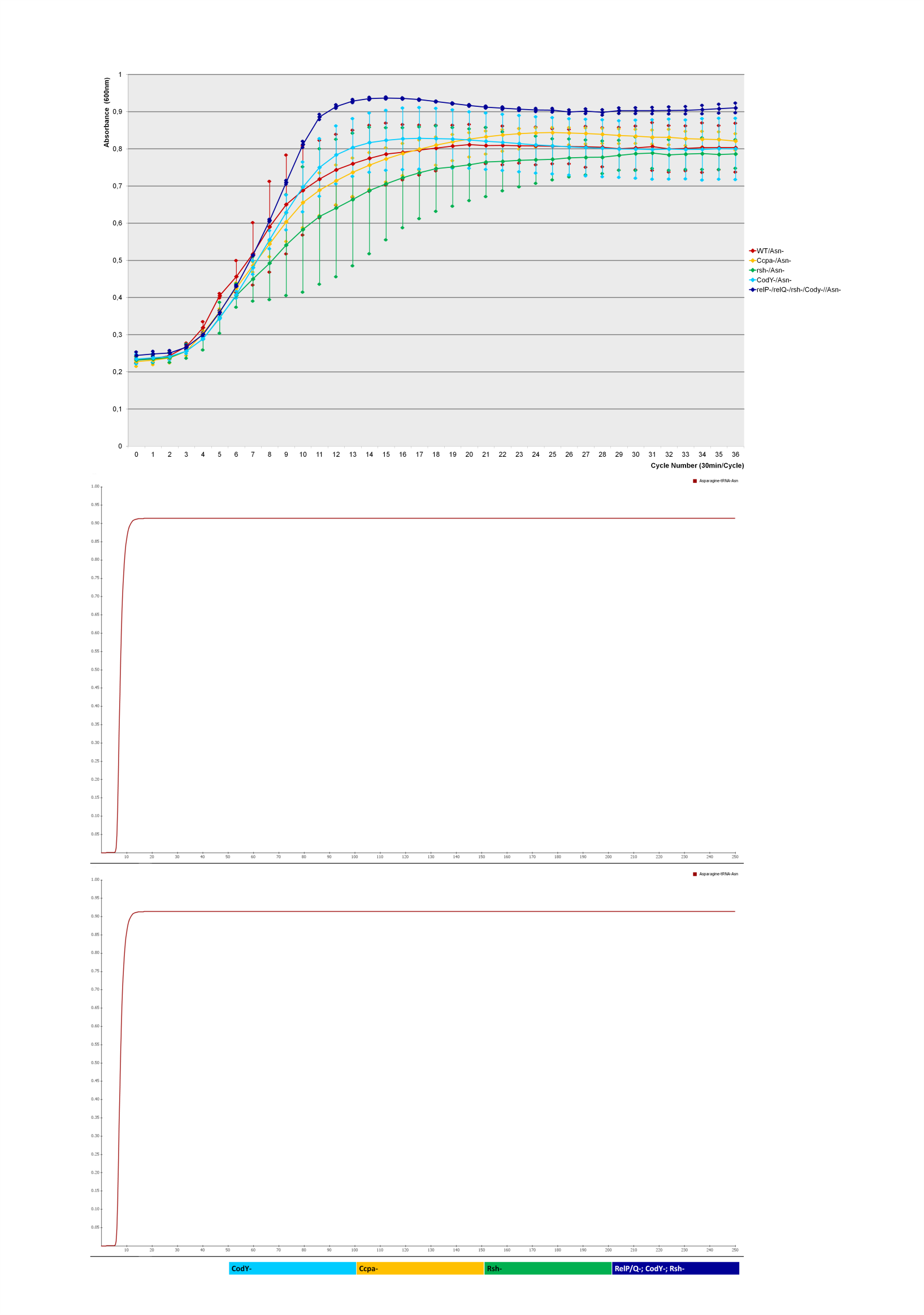


S1c) Asn-


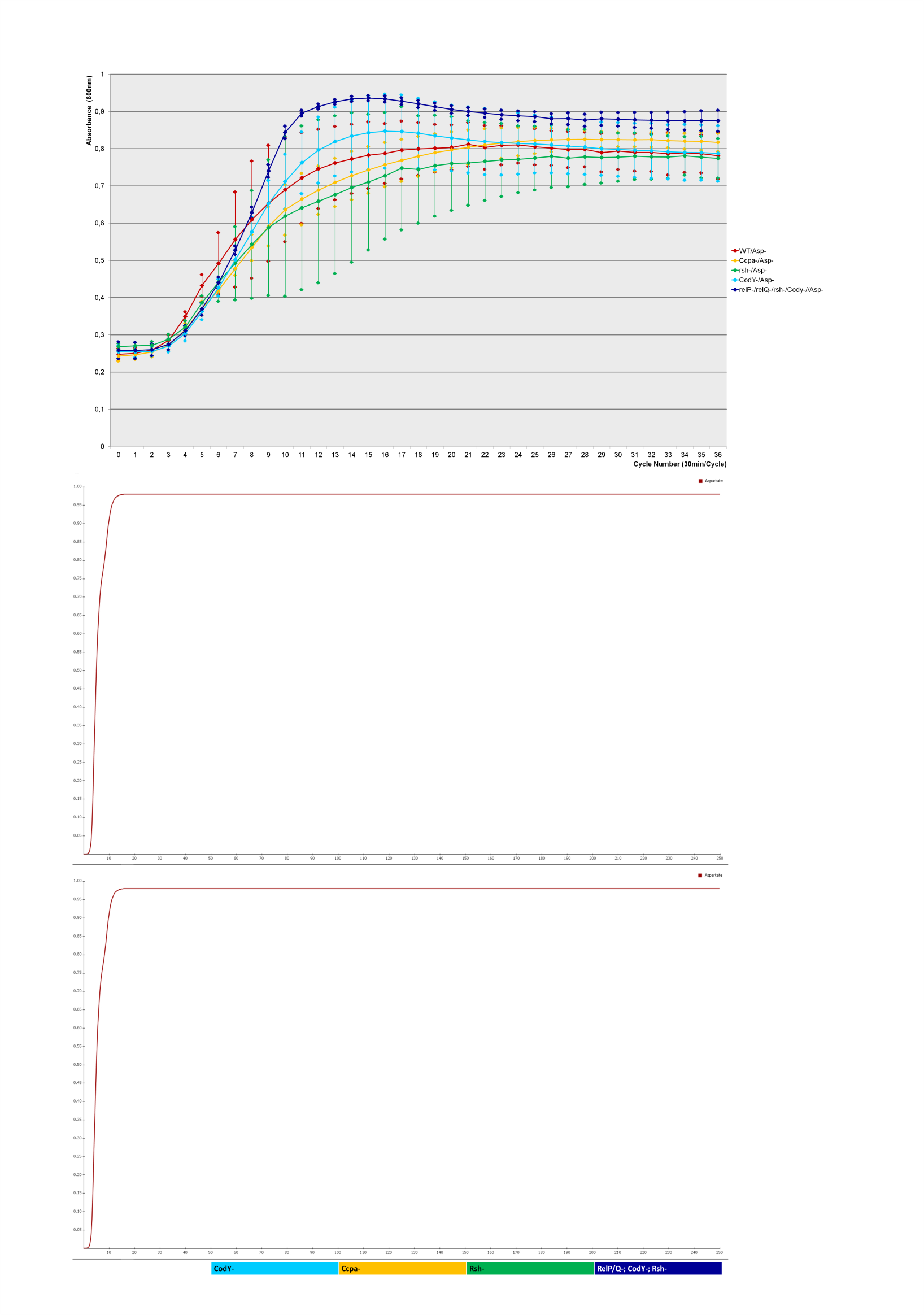


S1d) Asp-


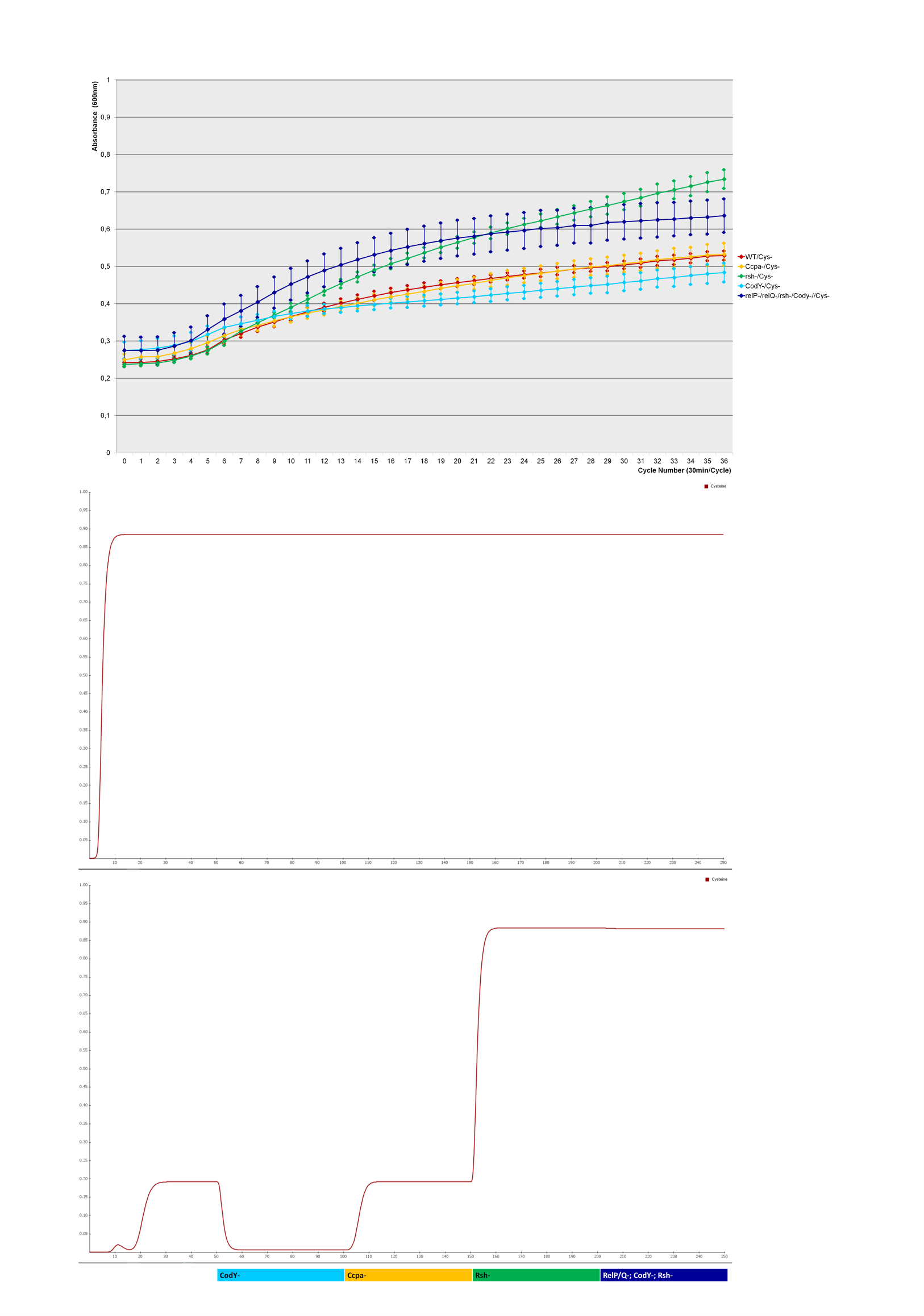


S1e) Cys-


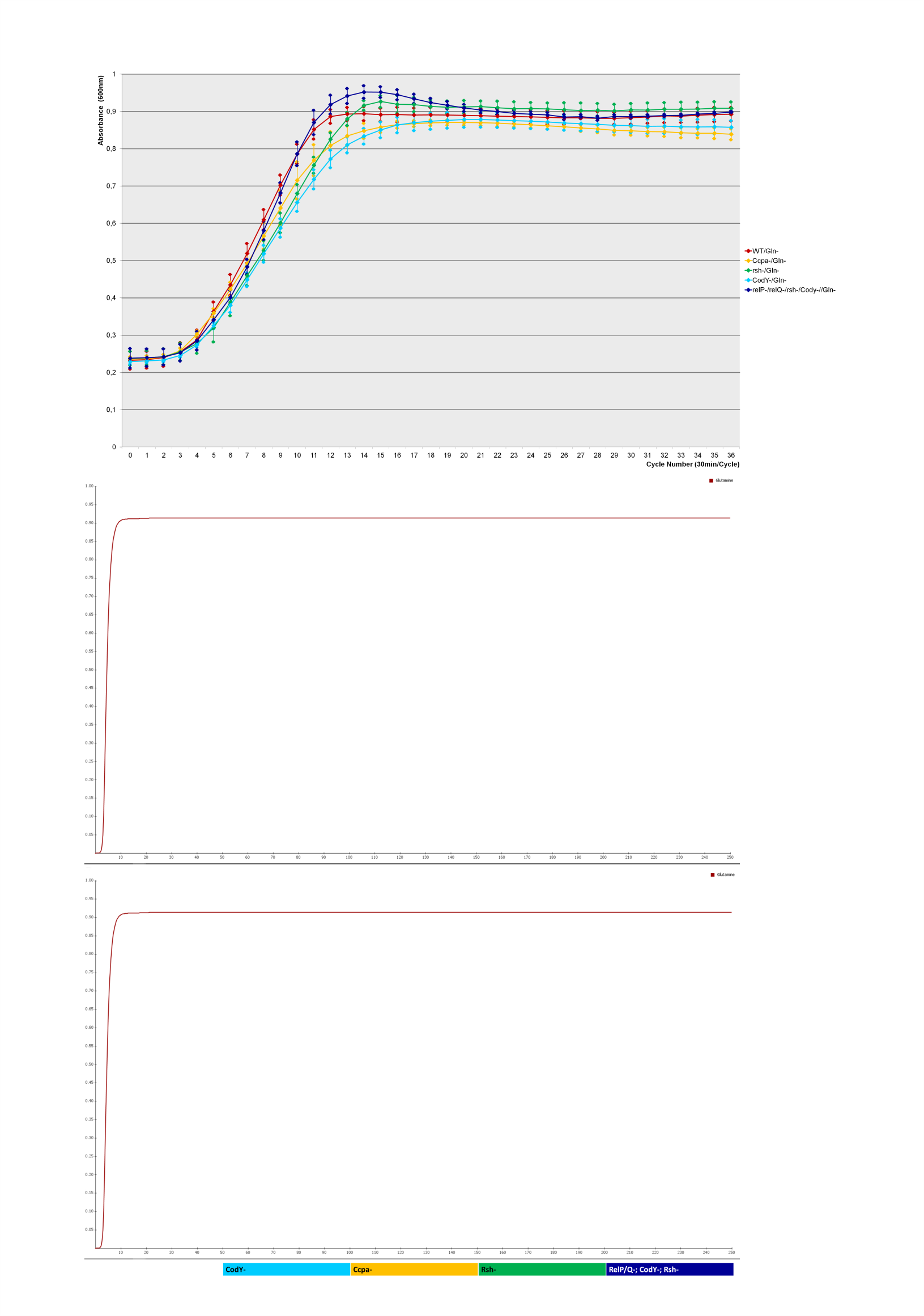


S1f) Gln-


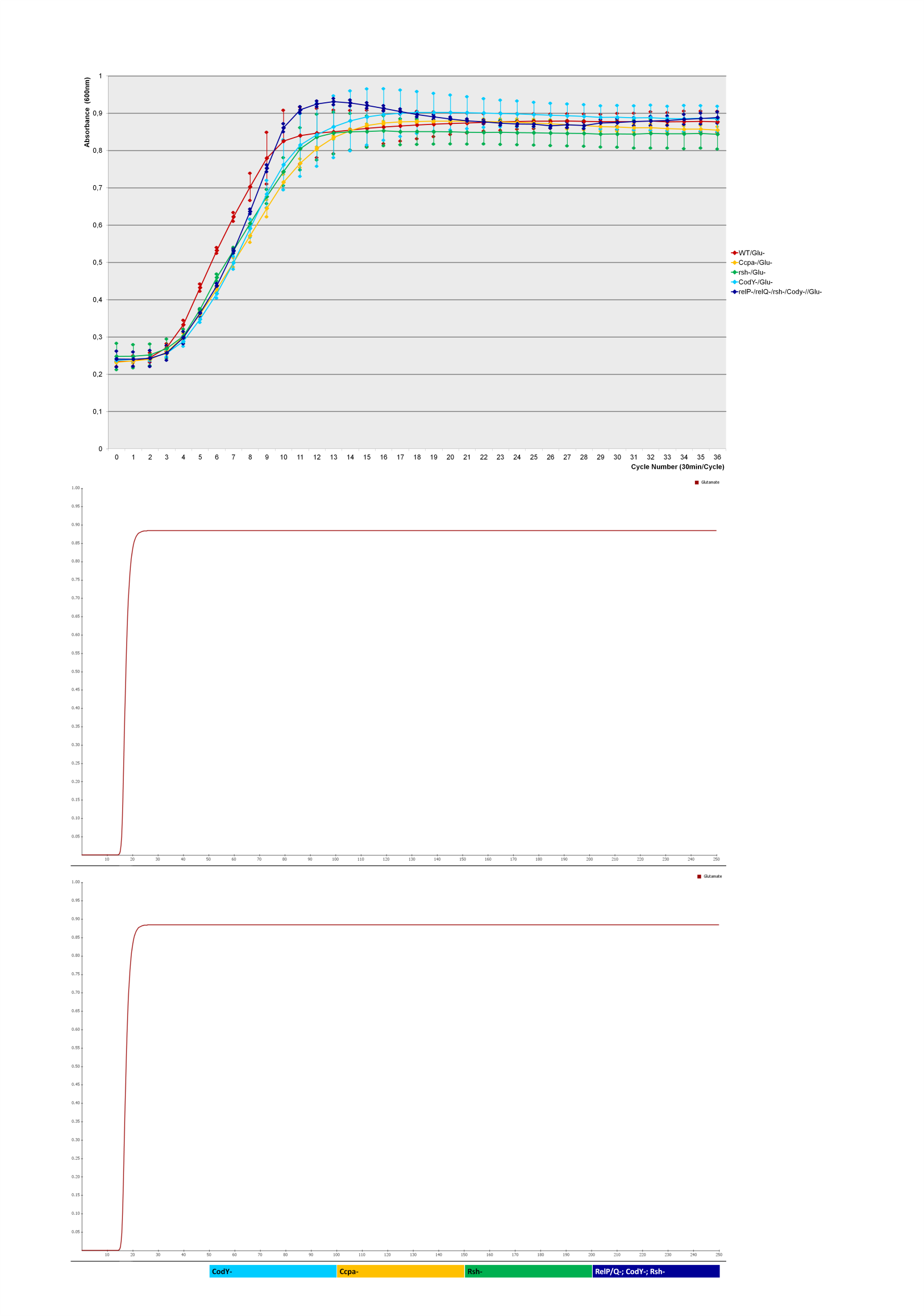


S1g) Glu-


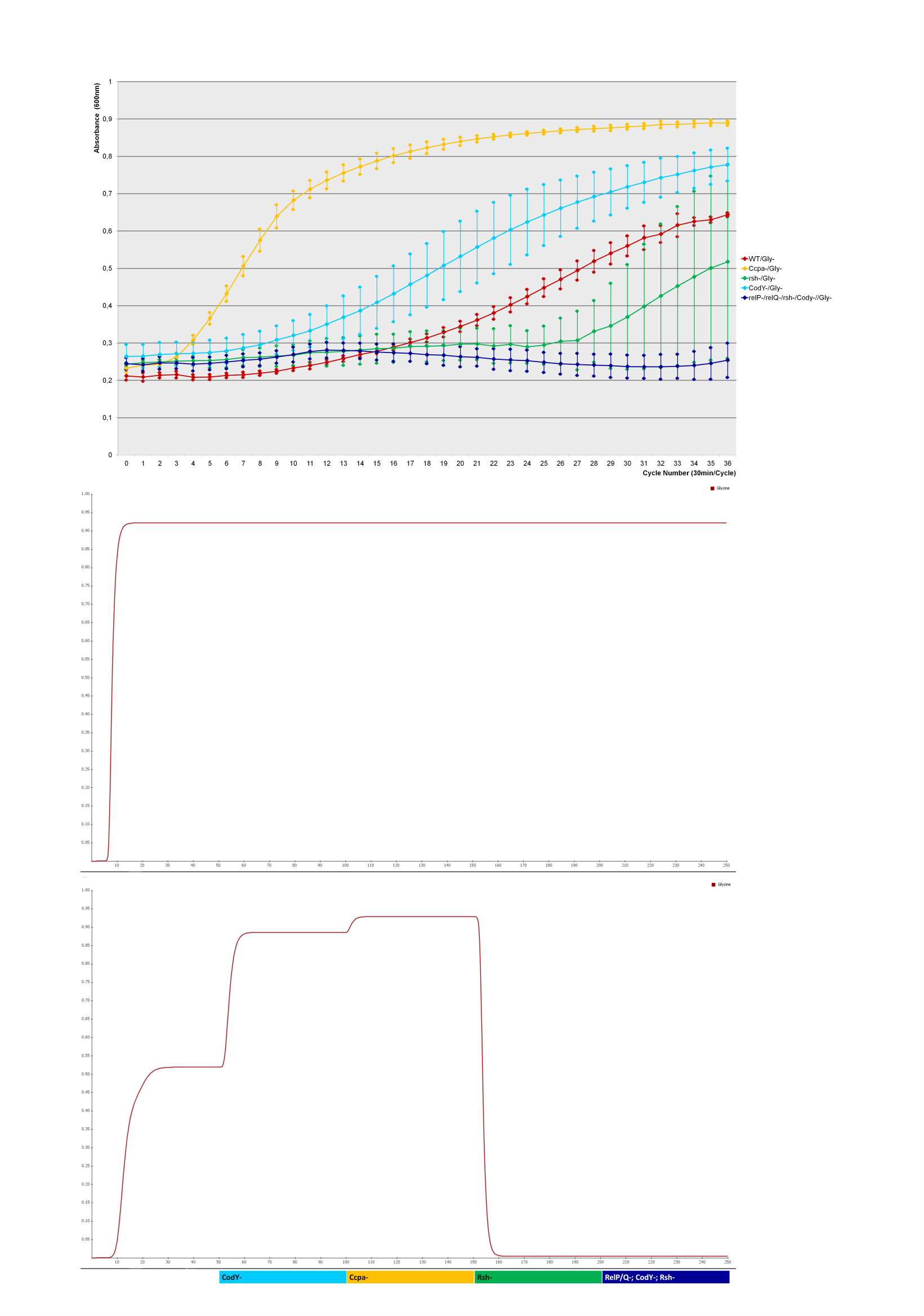


S1h) Gly-


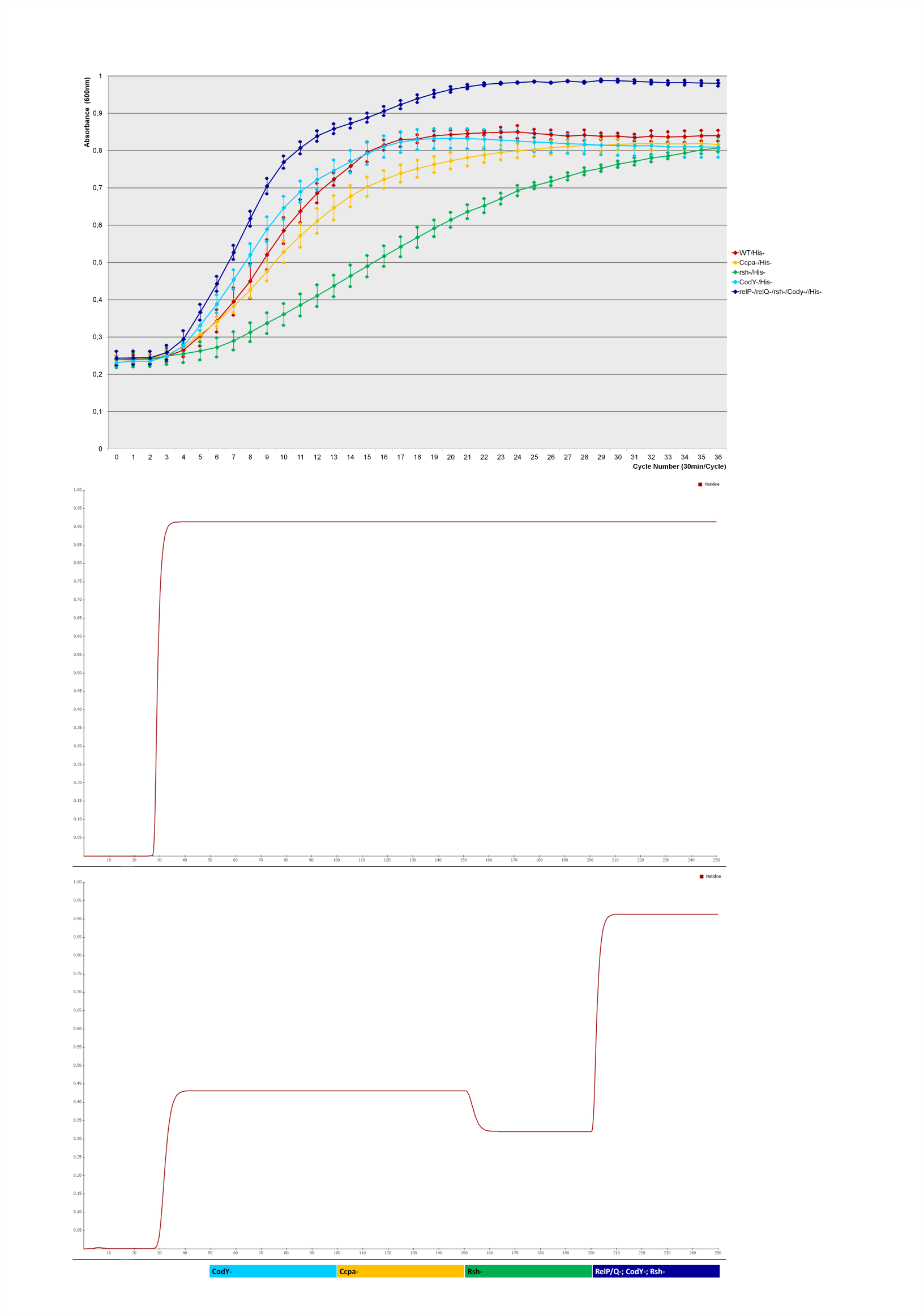


S1i) His-


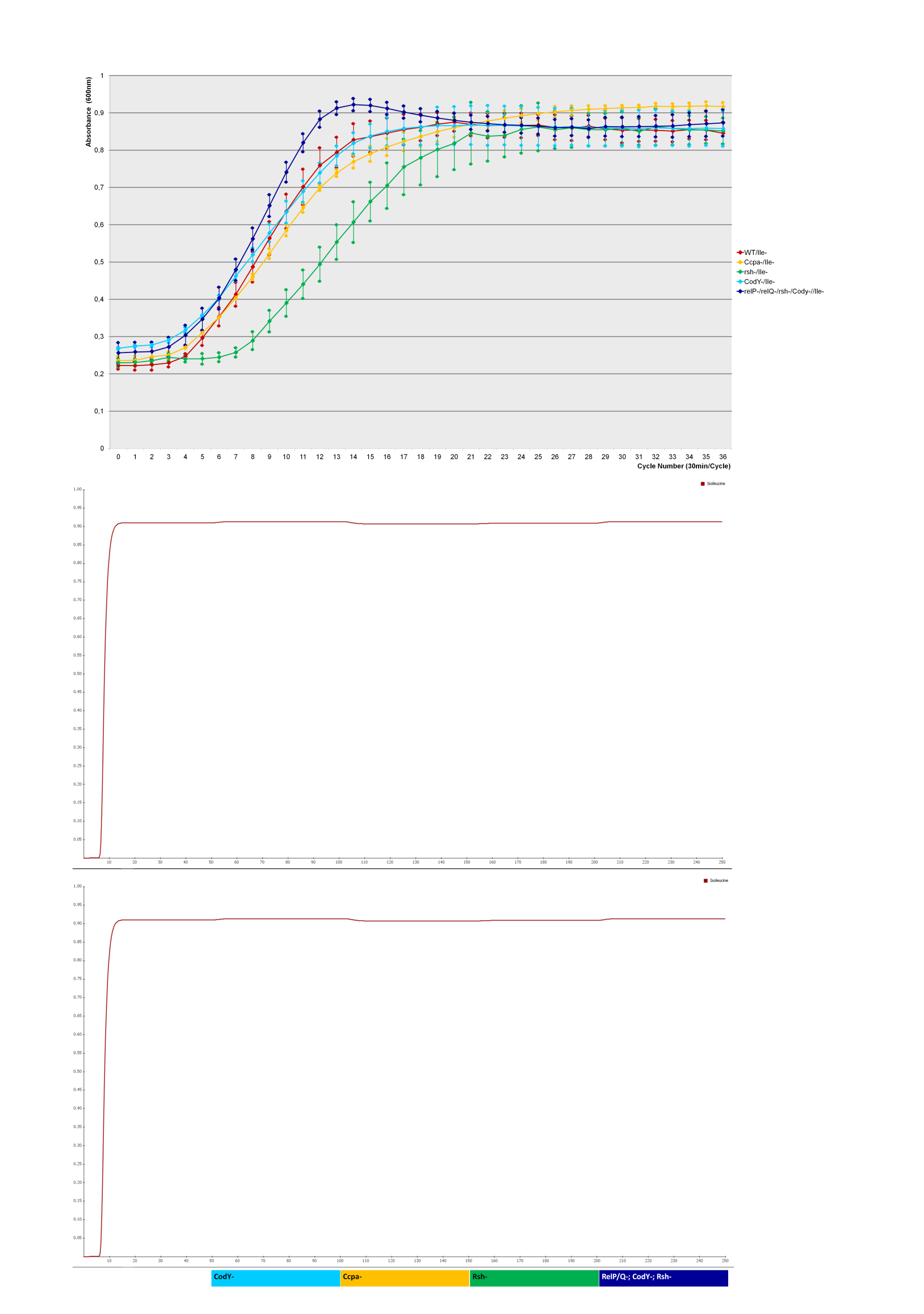


S1j) Ile-


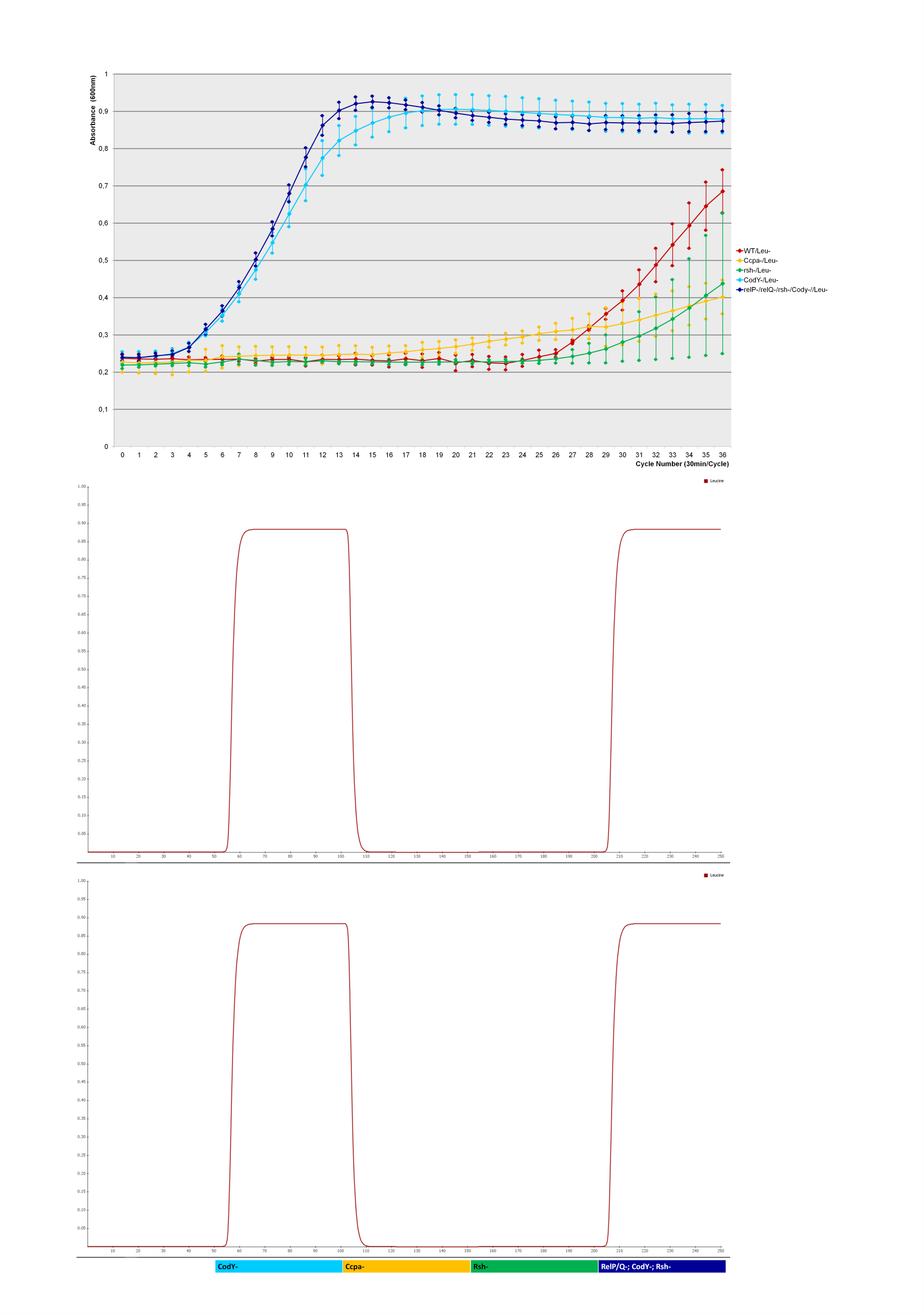


S1k) Leu-


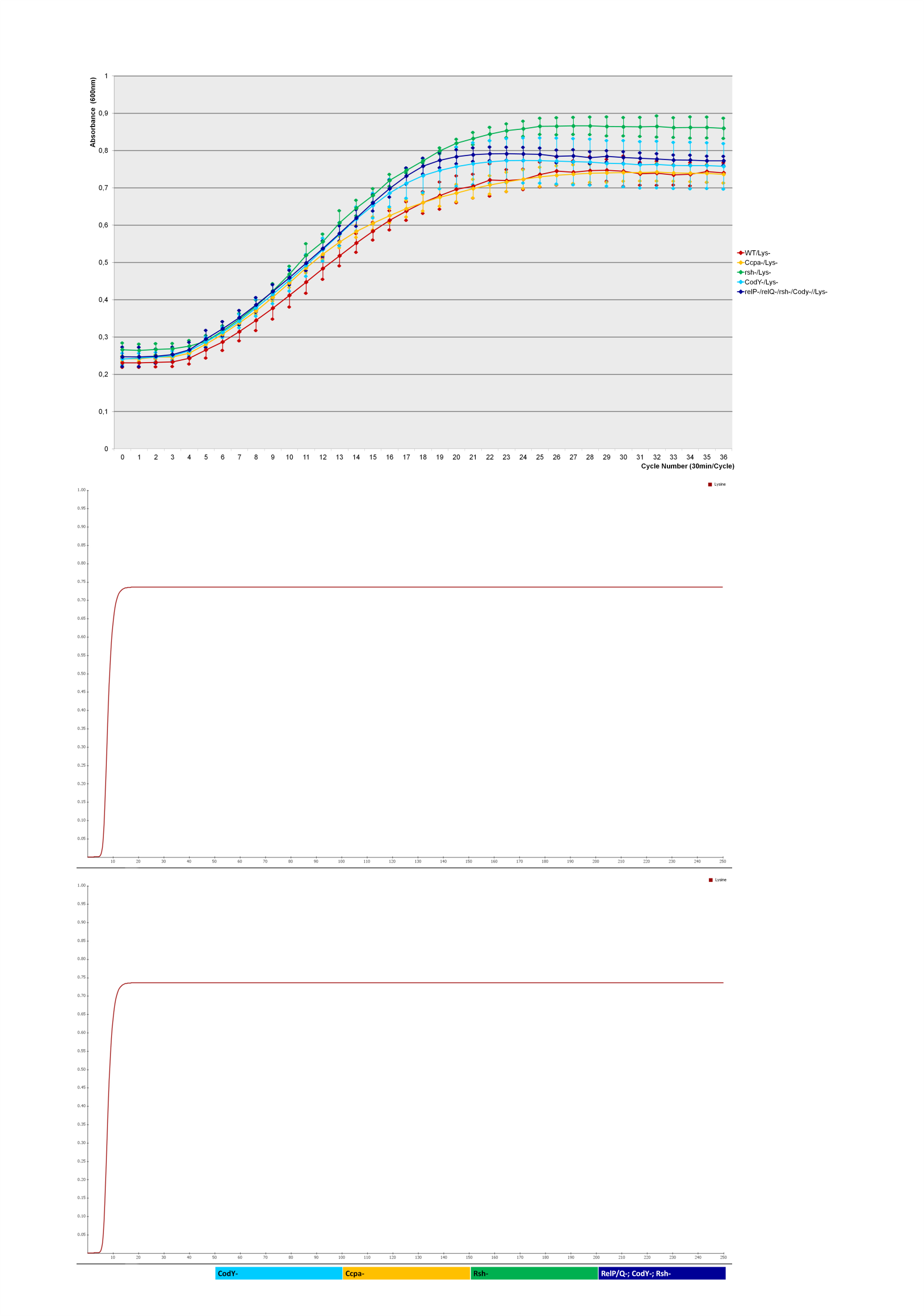


S1l) Lys-


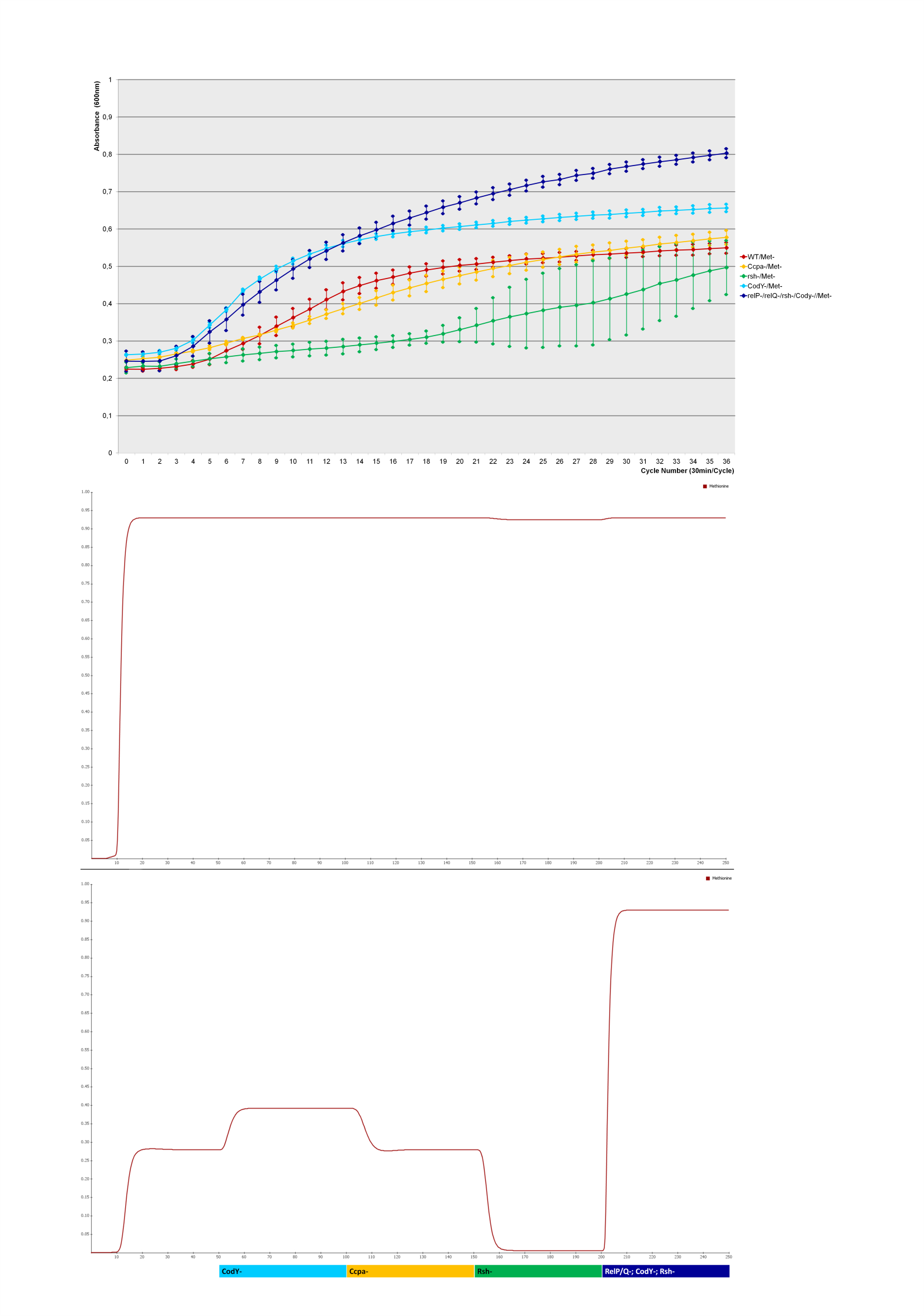


S1m) Met-


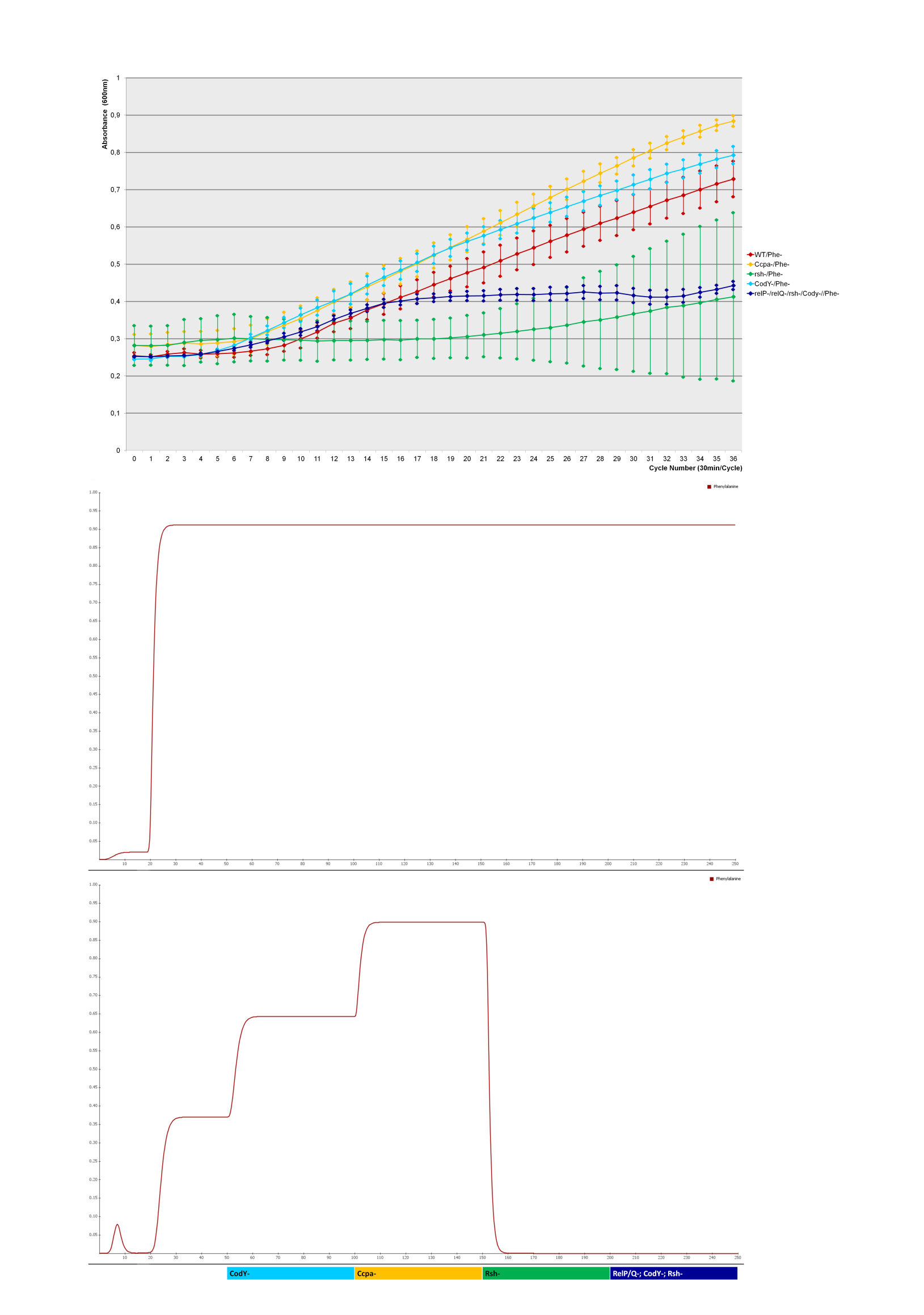


S1n) Phe-


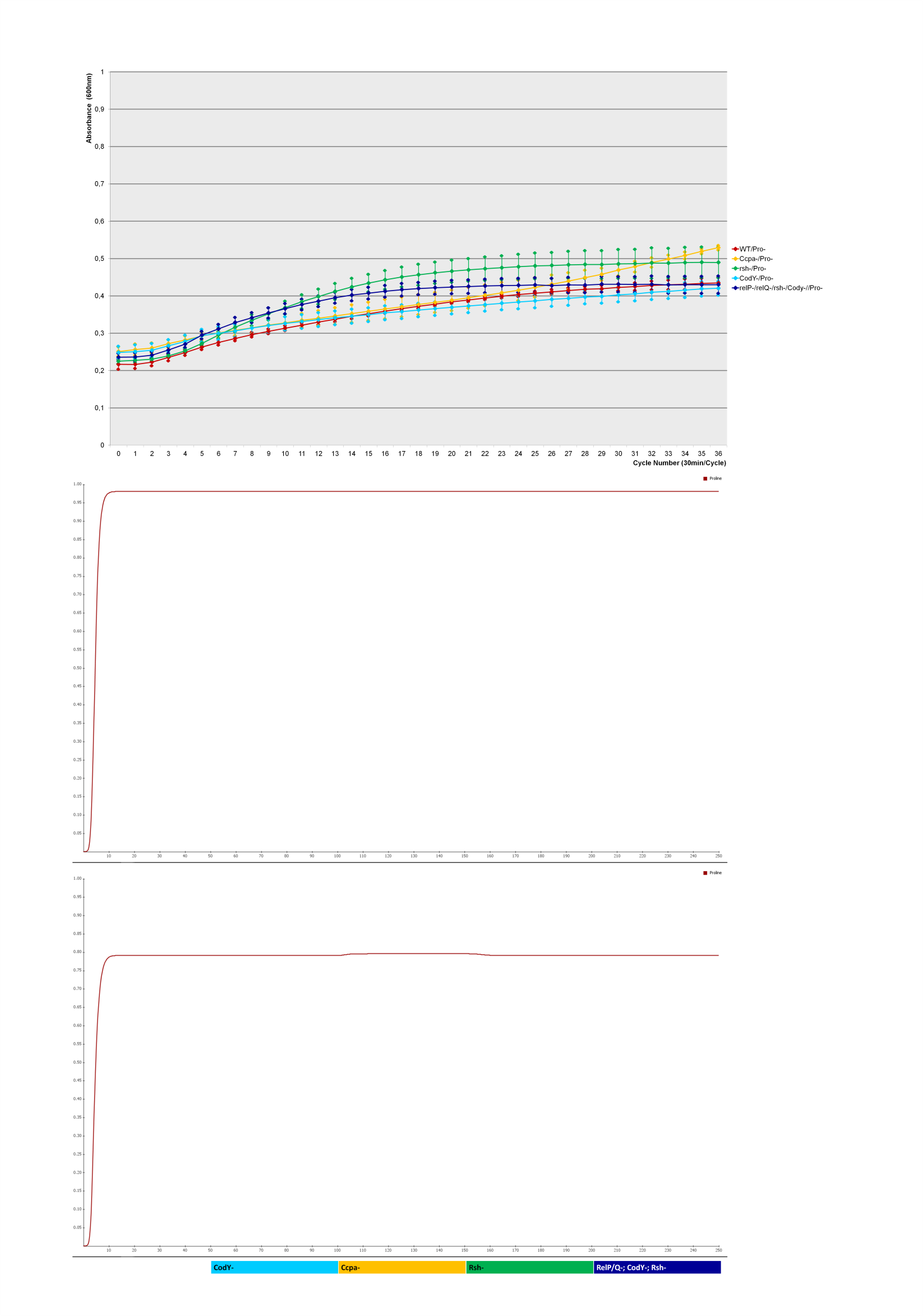


S1o) Pro-


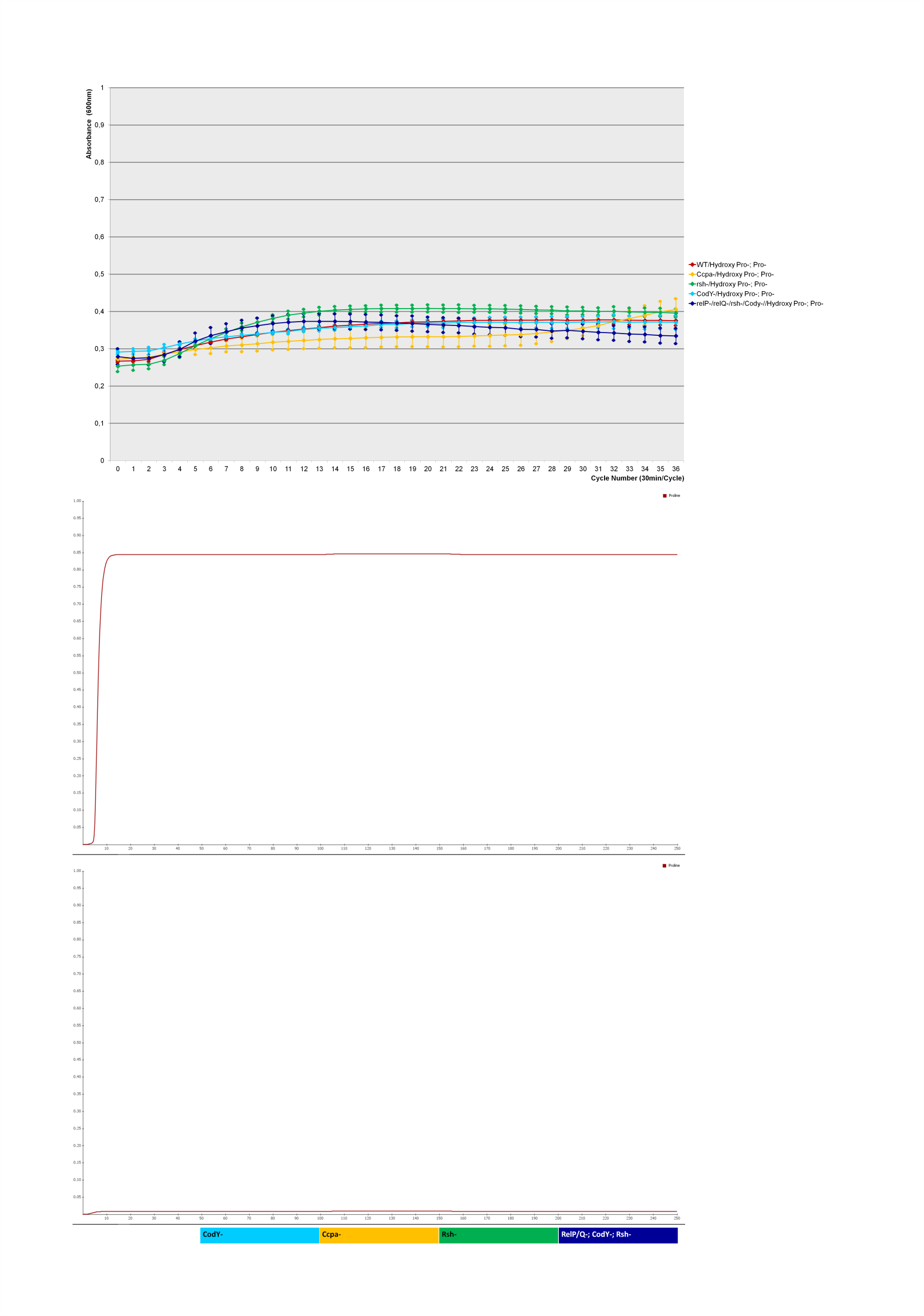


S1p) Hydroxy Pro-/Pro-


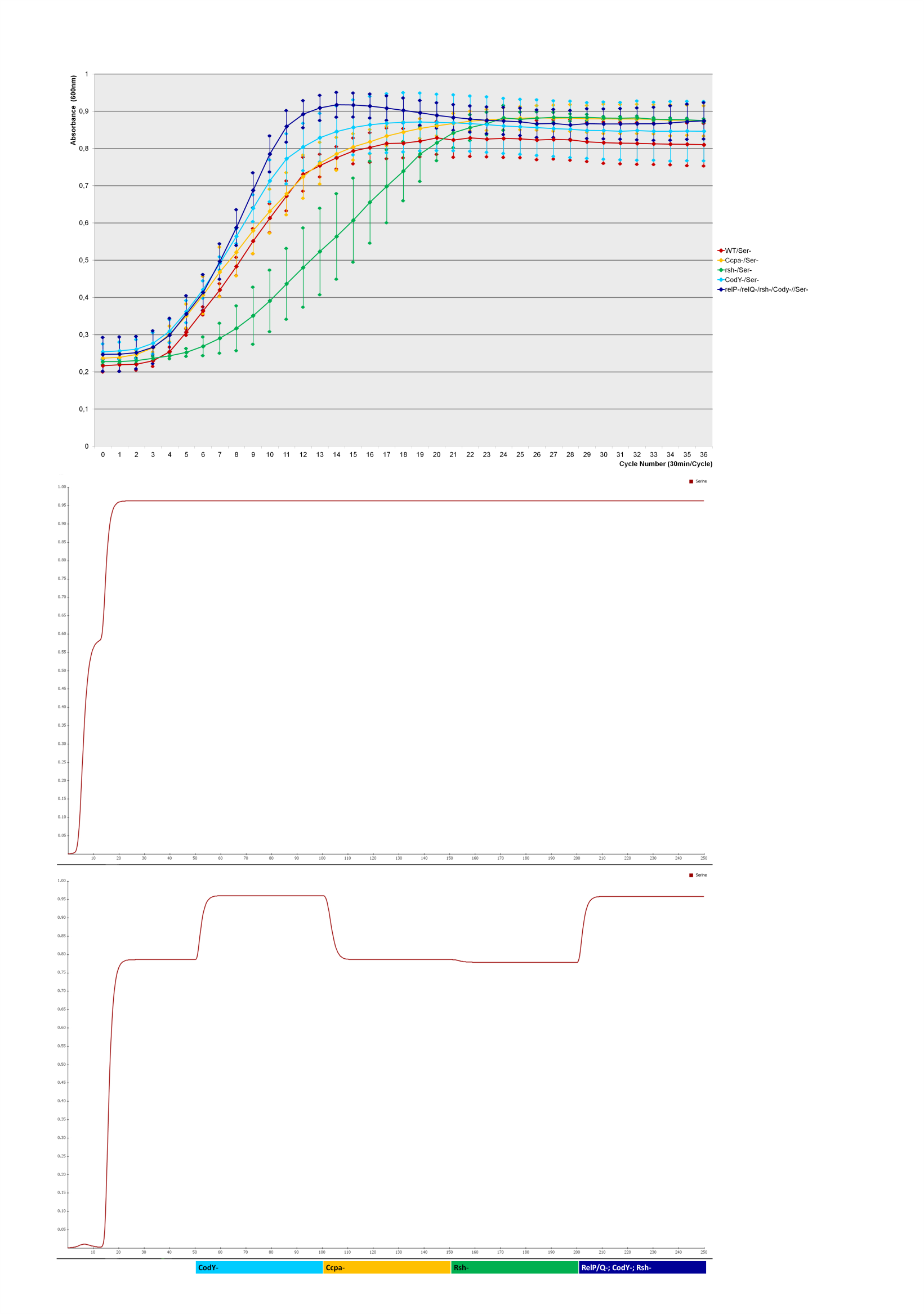


S1q) Ser-


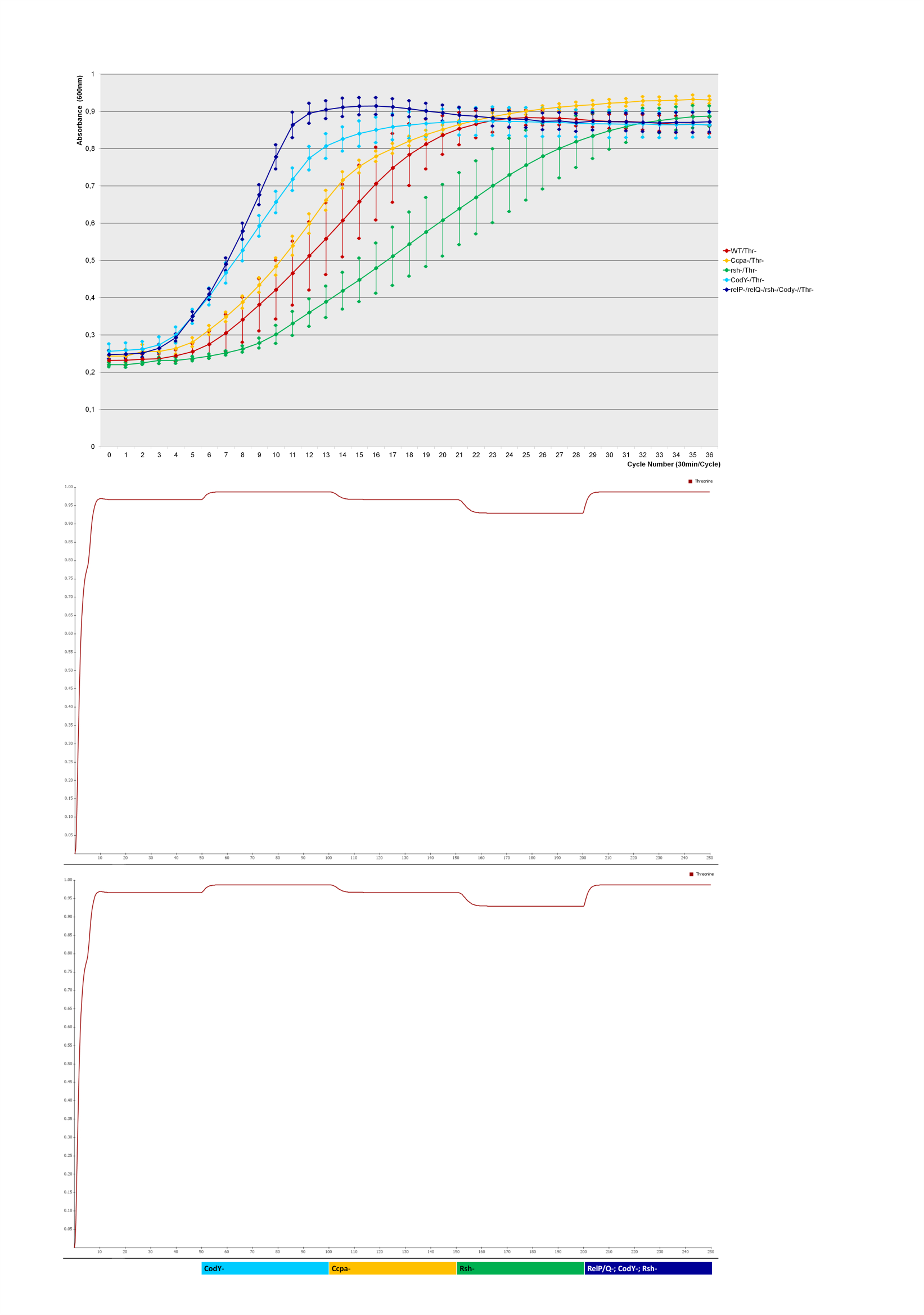


S1r) Thr-


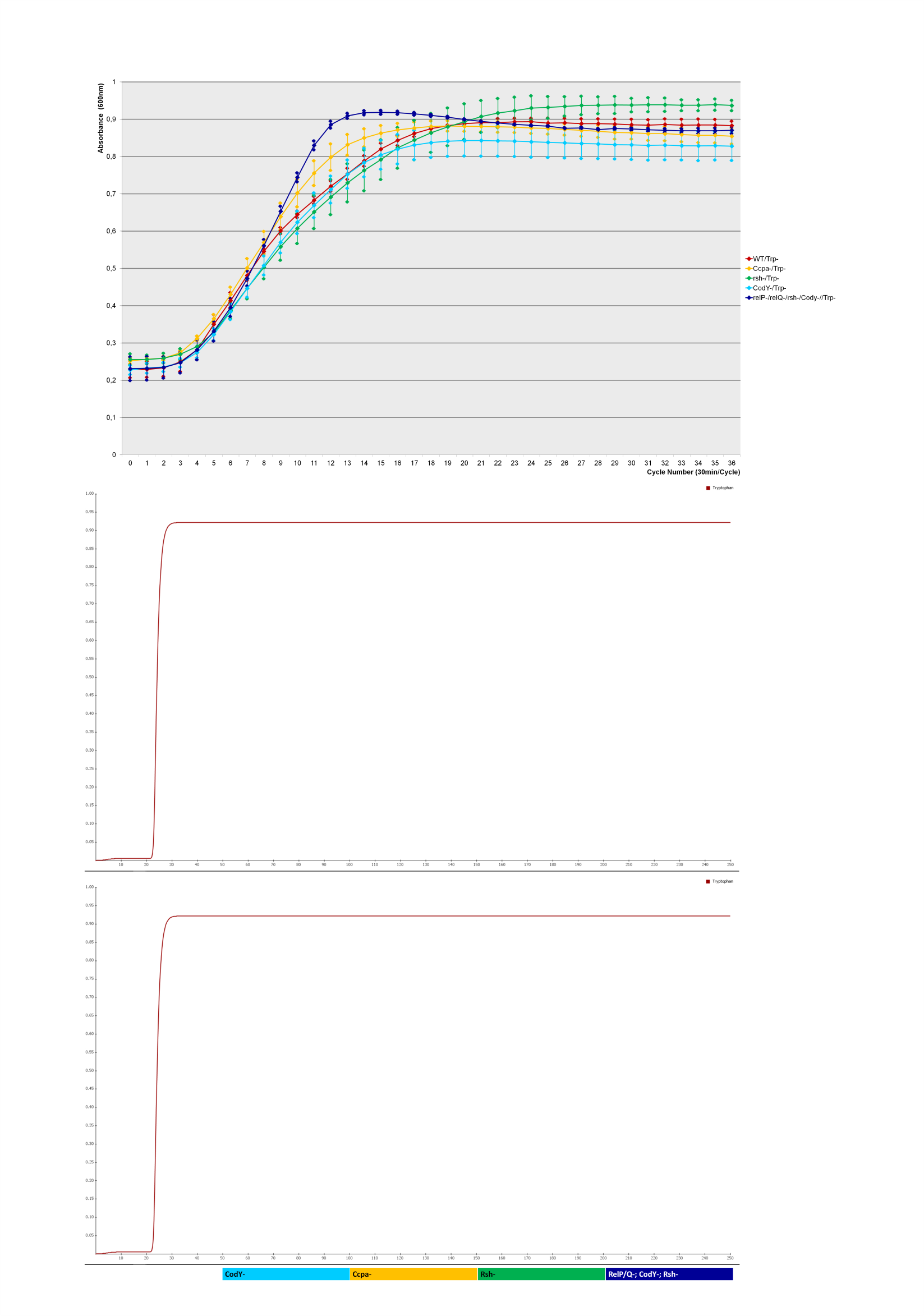


S1s) Trp-


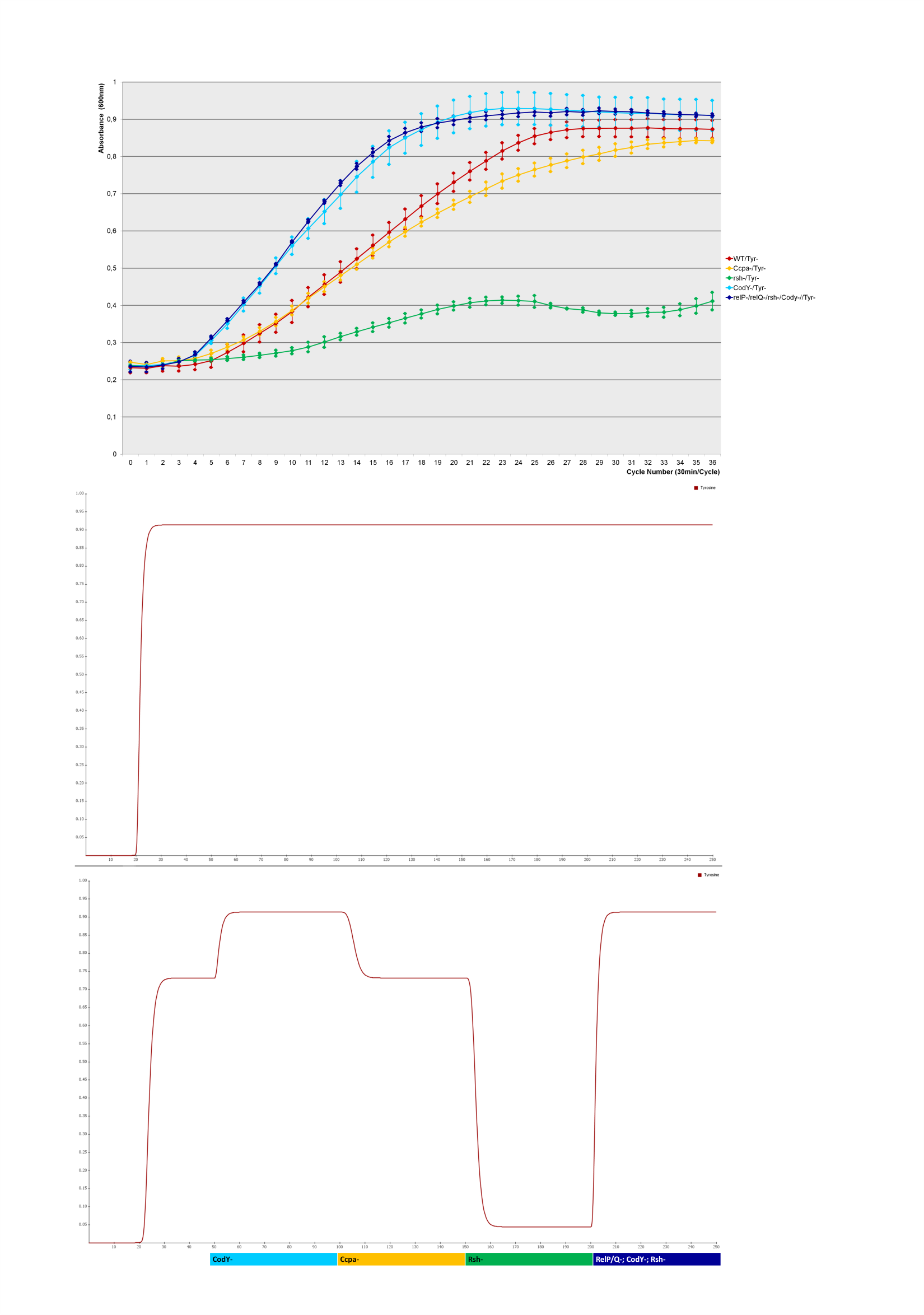


S1t) Tyr-


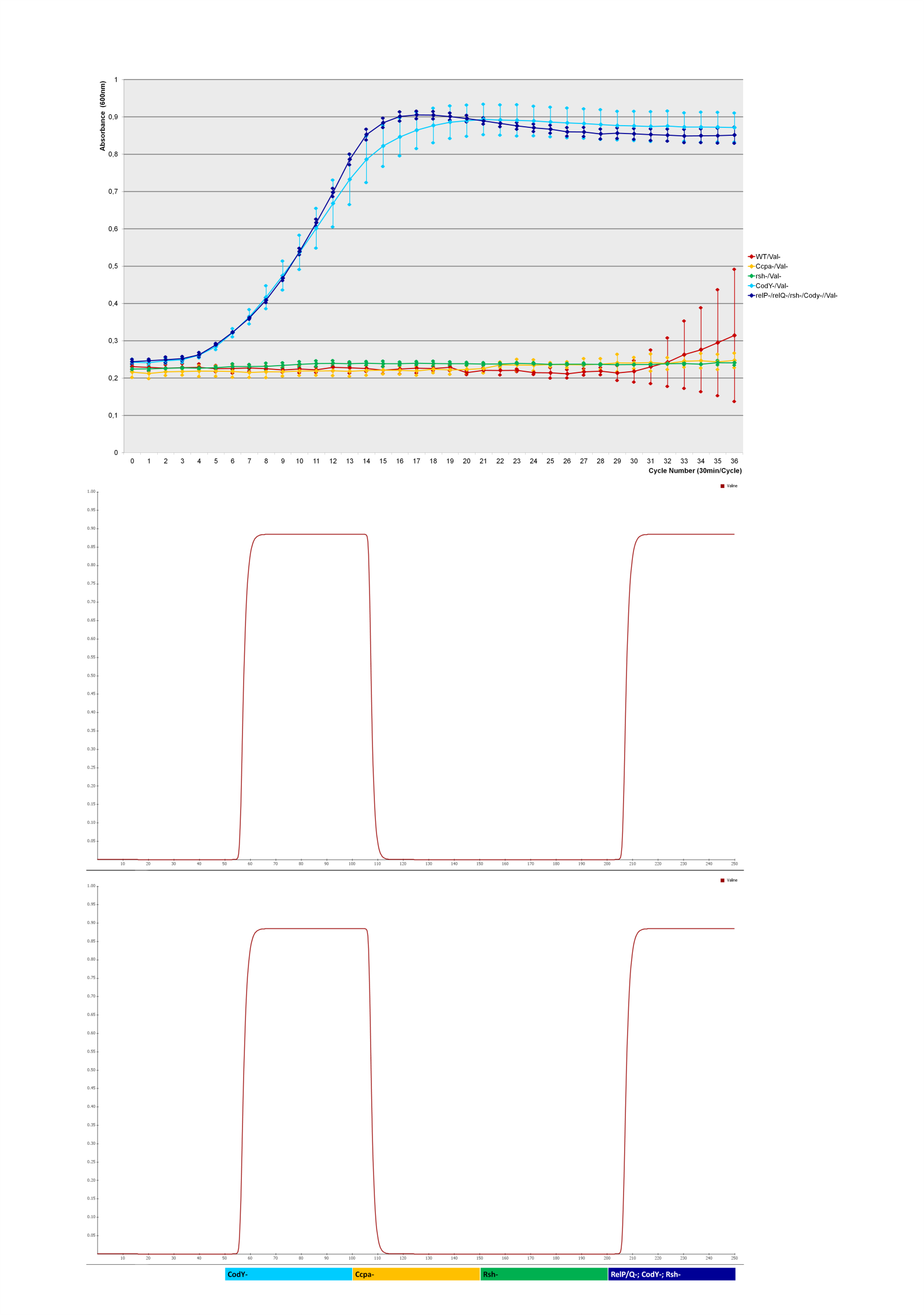


S1u) Val-

**Figure S2 a-e:**


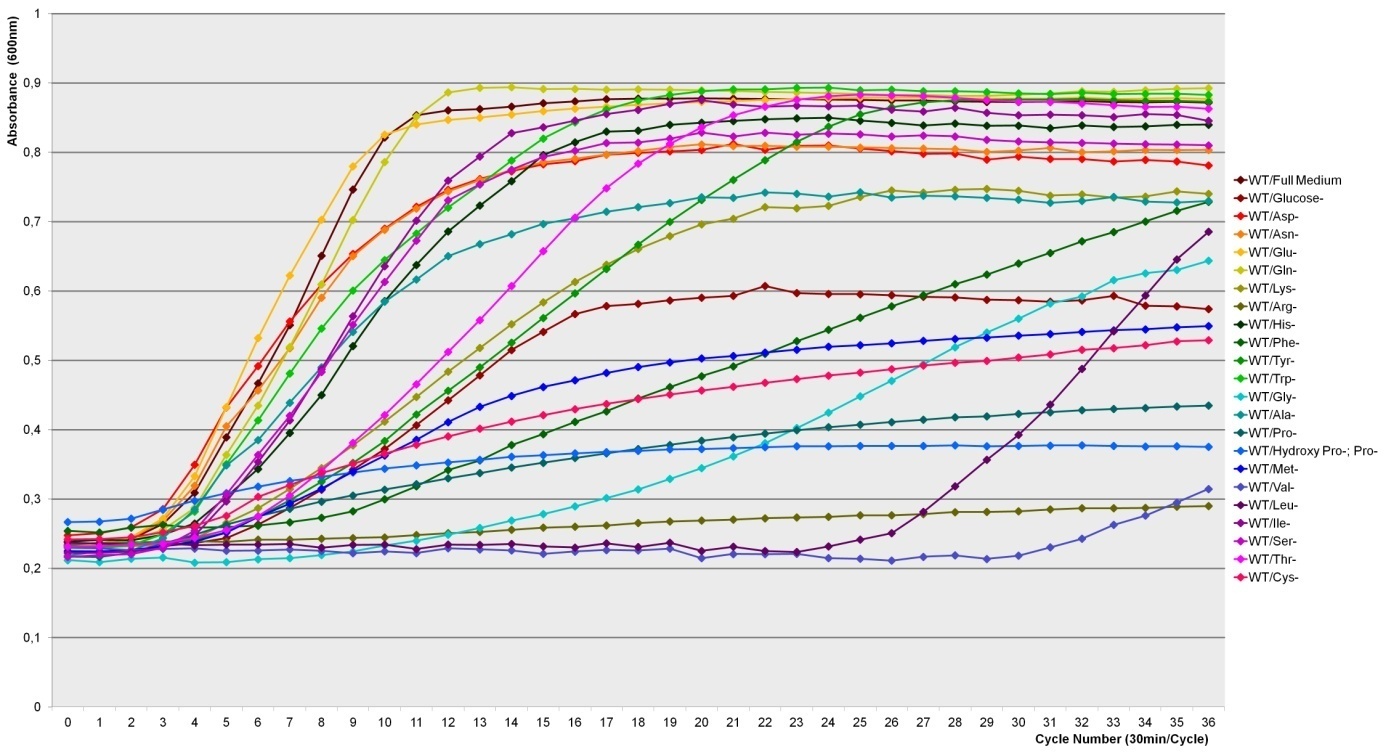
Figure S2a: WT


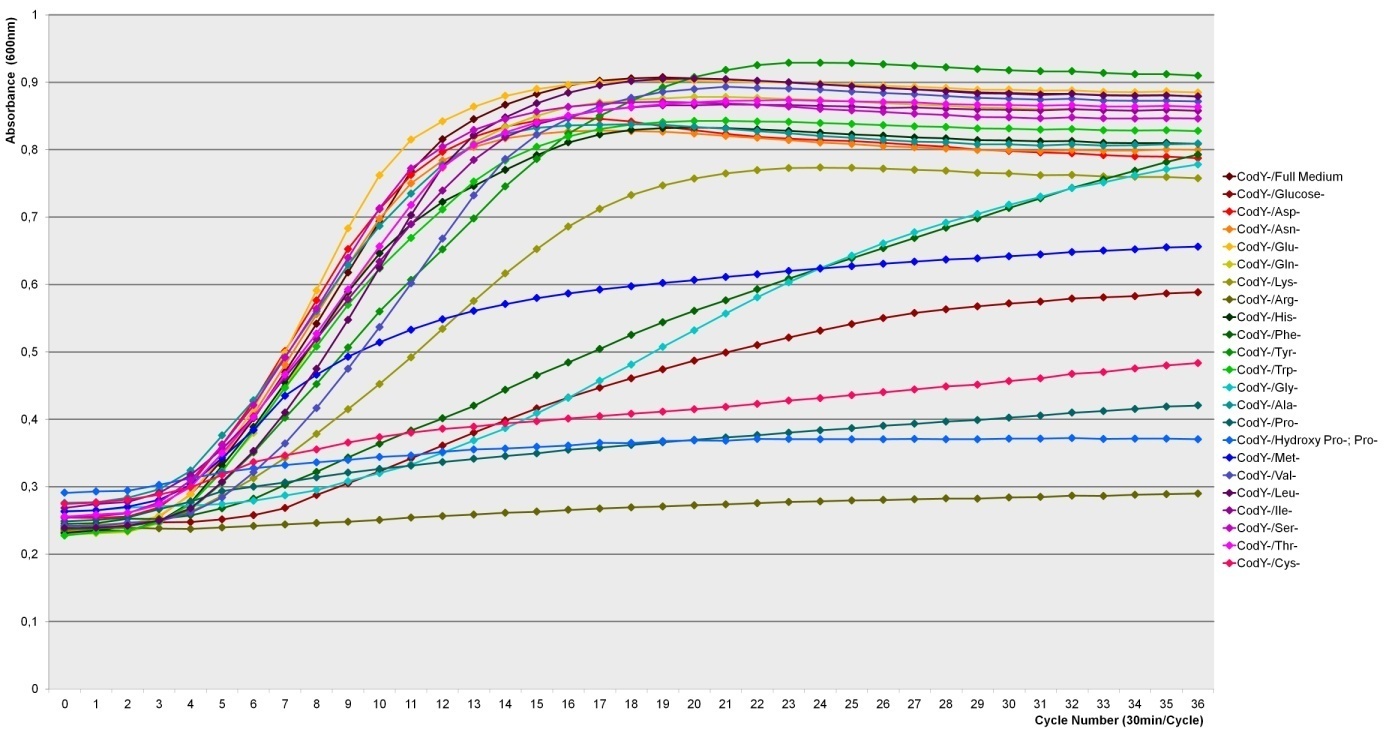
Figure S2b: Cody-


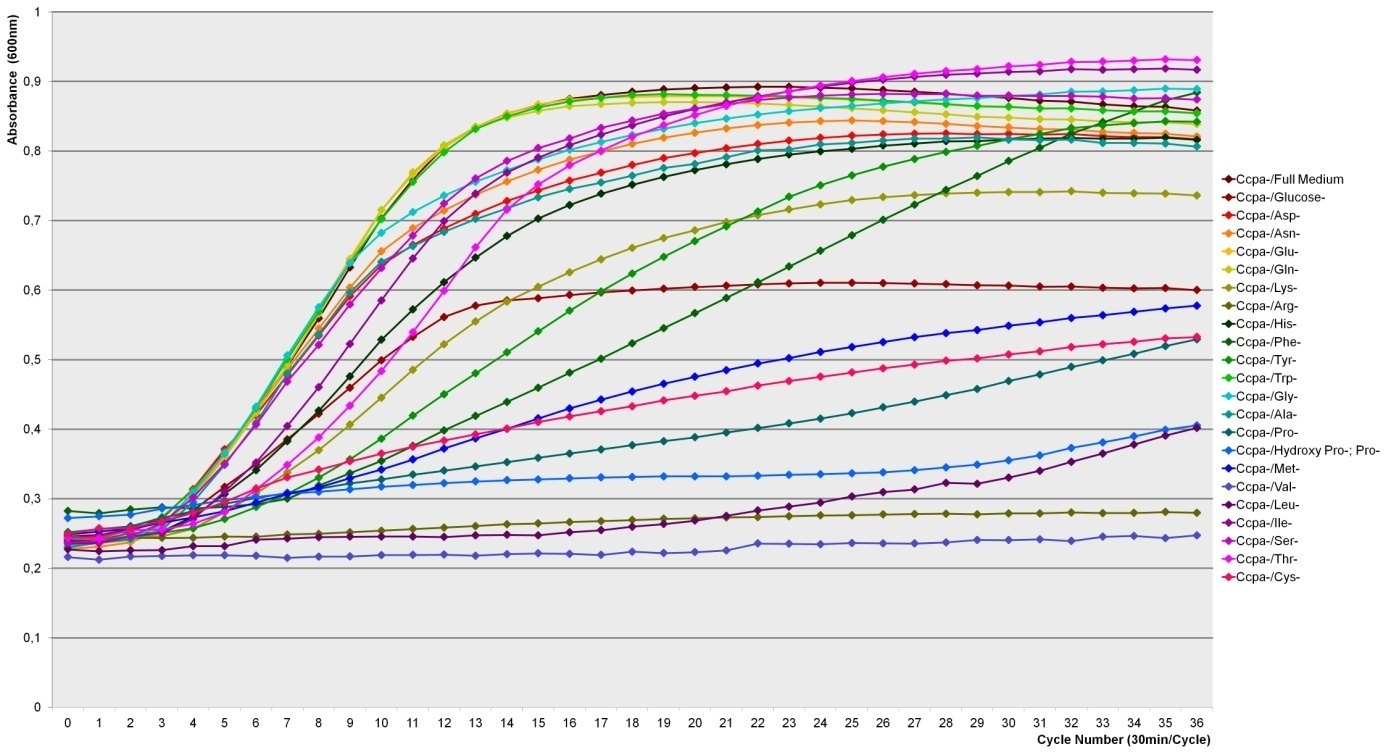
Figure S2c: Ccpa-


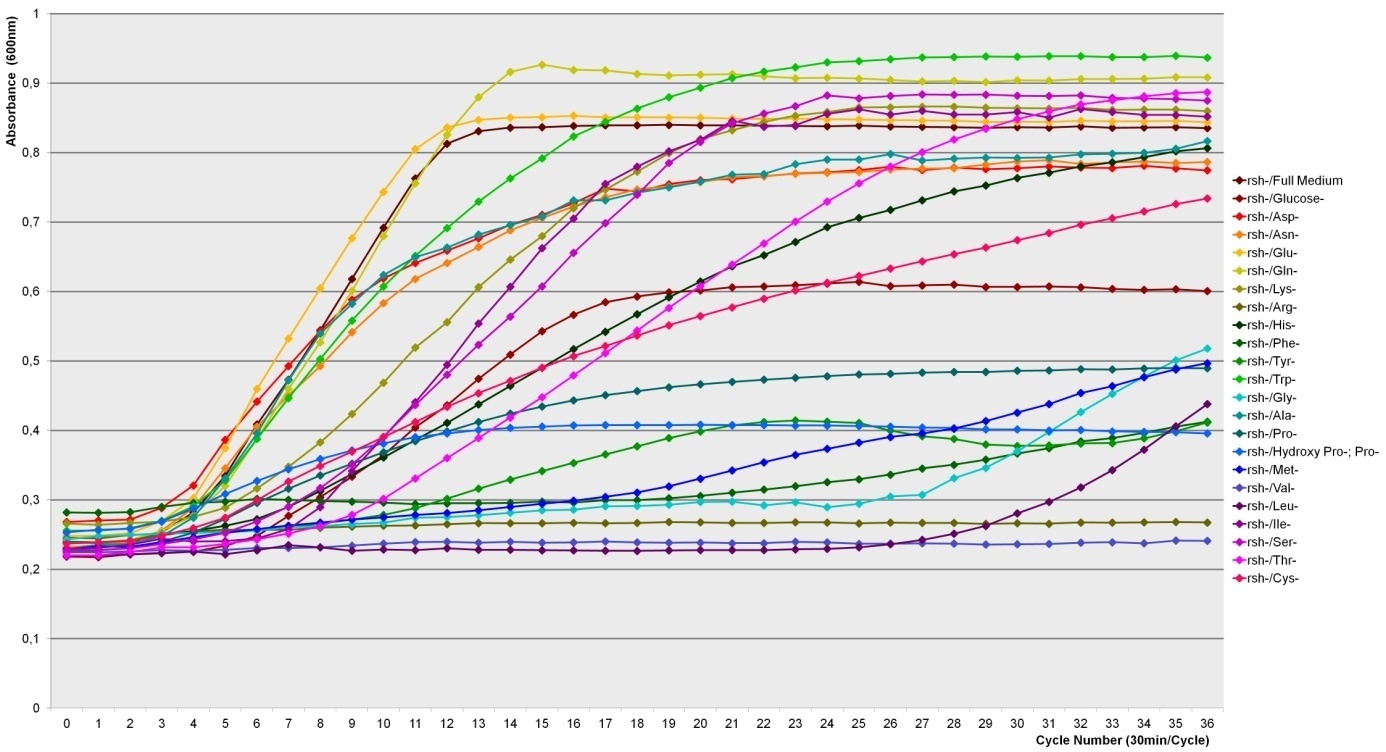
Figure S2d: Rsh-


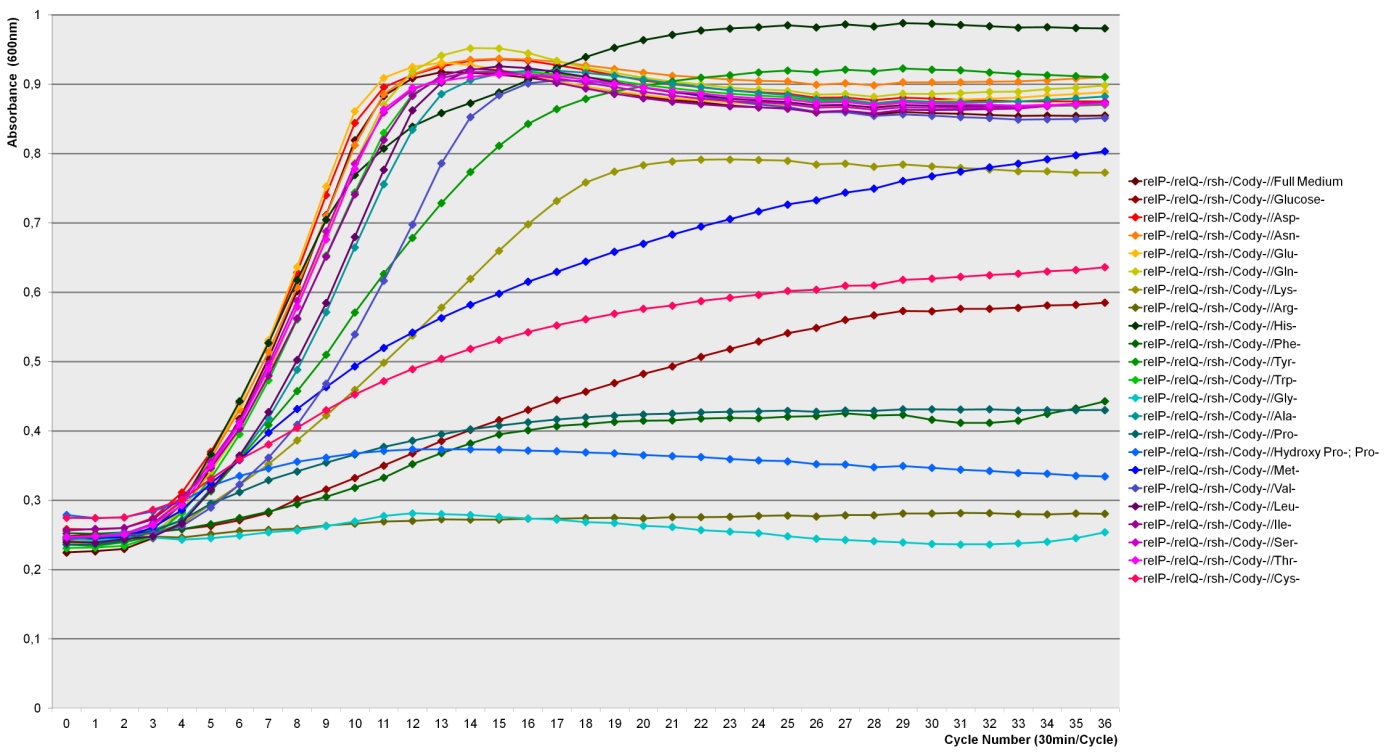
Figure S2e: RelP/Q-; rsh-; cody-

**Figure S3:**

**
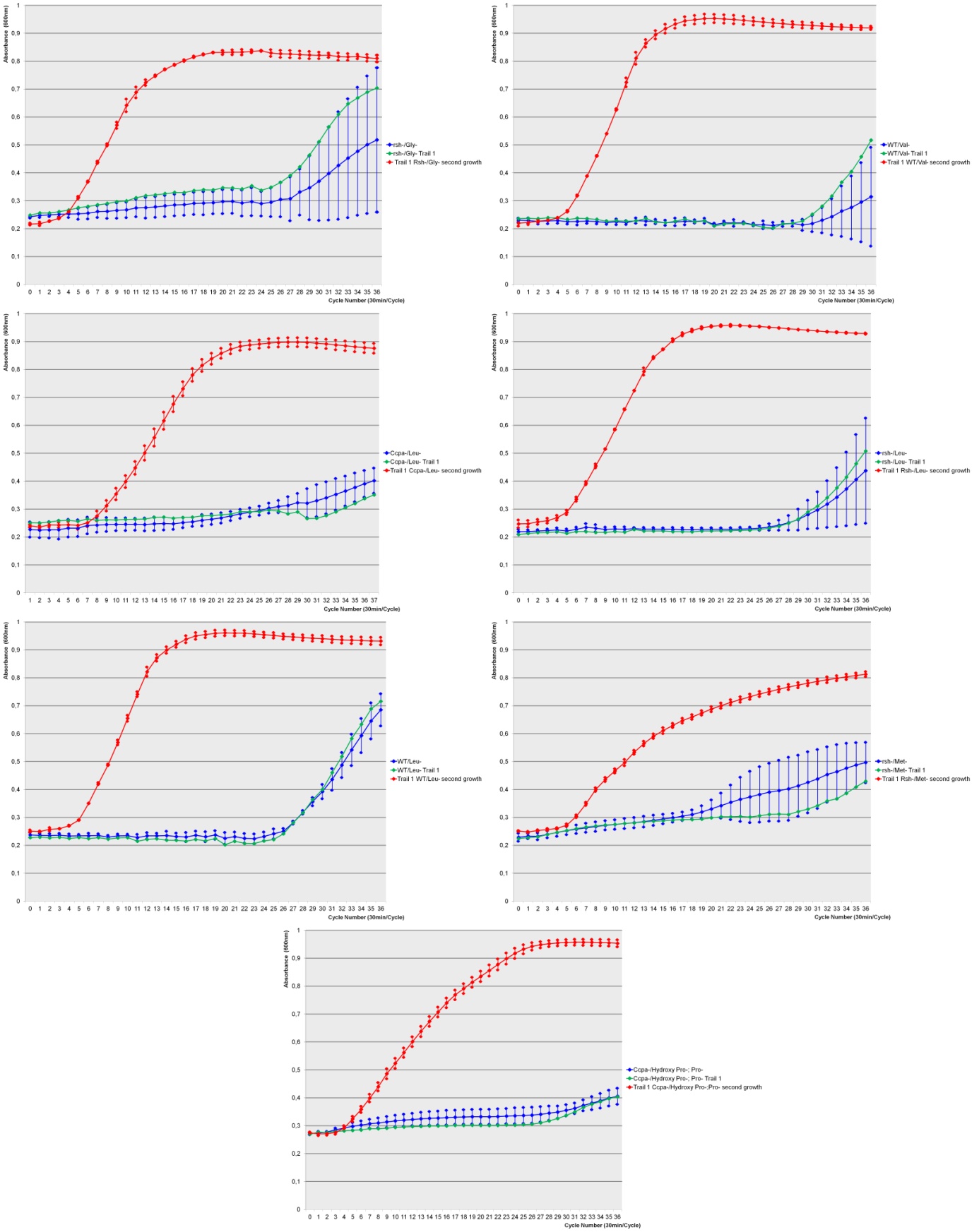
**

● CcpA/Hydroxy Pro-;Pro-
● CcpA/Hydroxy Pro-;Pro- trial 1
● Trial 1 CcpA/Hydroxy Pro-;Pro-
second growth

● rsh/Met-
● rsh/Met- trial 1
● Trial 1 rsh/Met-
second growth

● rsh/Leu-
● rsh/Leu- trial 1
● Trial 1 rsh/Leu-
second growth

● WT/Val-
● WT/Val- trial 1
● Trial 1 WT/Val-
second growth

● WT/Leu-
● WT/Leu- trial 1
● Trial 1 WT/Leu-
second growth

● CcpA/Leu-
● CcpA/Leu- trial 1
● Trial 1 WT/Leu-
second growth

● rsh/Gly-
● rsh/Gly- trial 1
● Trial 1 rsh/Gly-
second growth

**Figure S4:**


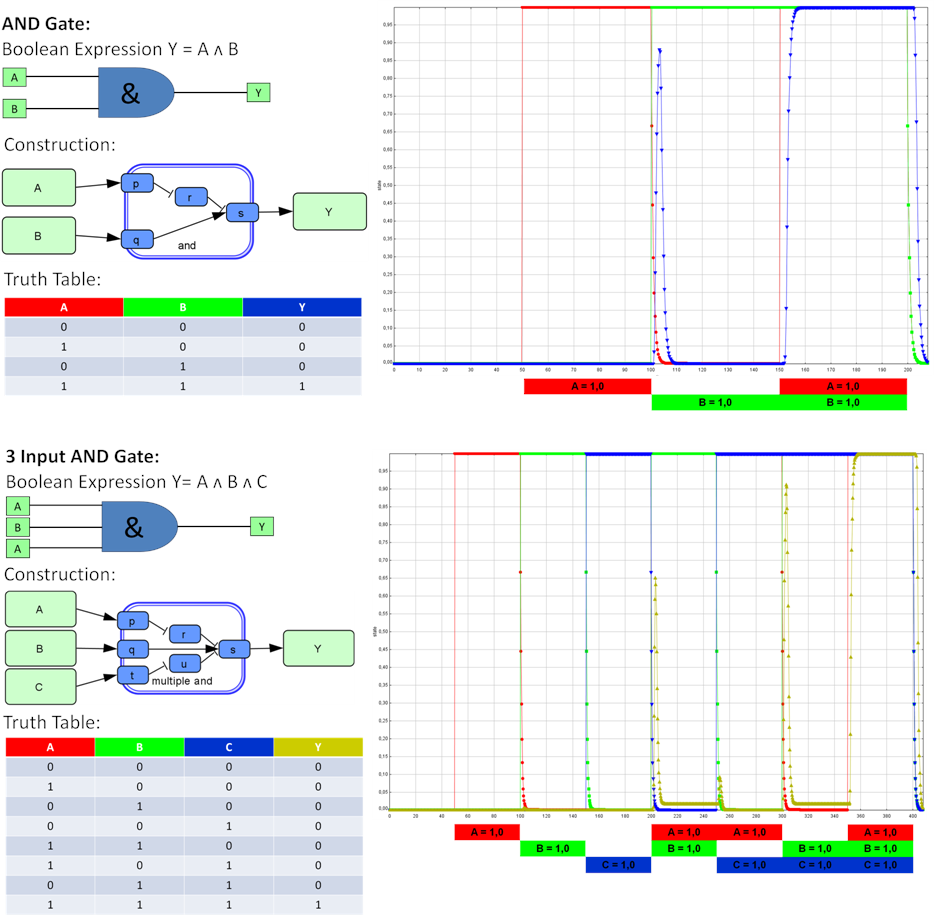


References

[1] A. L. Cheung, A. S. Bayer, G. Zhang et al., “Regulation of virulence determinants in vitro and in vivo in Staphylococcus aureus,” *FEMS Immunology & Medical Microbiology*, vol. 40, no. 1, pp. 1–9, 2004.

[2] C. Audretsch, D. Lopez, M. Srivastava et al., “A semi-quantitative model of Quorum-Sensing in Staphylococcus aureus, approved by microarray meta-analyses and tested by mutation studies,” *Molecular bioSystems*, vol. 9, no. 11, pp. 2665–2680, 2013.

[3] R. Cassels, B. Oliva, and D. Knowles, “Occurrence of the regulatory nucleotides ppGpp and pppGpp following induction of the stringent response in staphylococci,” *Journal of Bacteriology*, vol. 177, no. 17, pp. 5161–5165, 1995.

[4] C. D. Majerczyk, P. M. Dunman, T. T. Luong et al., “Direct targets of CodY in Staphylococcus aureus,” *Journal of Bacteriology*, vol. 192, no. 11, pp. 2861–2877, 2010.

[5] K. Seidl, S. Müller, P. François et al., “Effect of a glucose impulse on the CcpA regulon in Staphylococcus aureus,” *BMC microbiology*, vol. 9, p. 95, 2009.

[6] T. Geiger, C. Goerke, M. Fritz et al., “Role of the (p)ppGpp synthase RSH, a RelA/SpoT homolog, in stringent response and virulence of Staphylococcus aureus,” *Infection and immunity*, vol. 78, no. 5, pp. 1873–1883, 2010.

[7] S. Reiss, J. Pané-Farré, S. Fuchs et al., “Global analysis of the Staphylococcus aureus response to mupirocin,” *Antimicrobial agents and chemotherapy*, vol. 56, no. 2, pp. 787–804, 2012.

[8] T. Geiger, P. Francois, M. Liebeke et al., “The stringent response of Staphylococcus aureus and its impact on survival after phagocytosis through the induction of intracellular PSMs expression,” *PLoS pathogens*, vol. 8, no. 11, e1003016, 2012.

[9] R. U. Rymer, F. A. Solorio, A. K. Tehranchi et al., “Binding mechanism of metal⋅NTP substrates and stringent-response alarmones to bacterial DnaG-type primases,” *Structure (London, England : 1993)*, vol. 20, no. 9, pp. 1478–1489, 2012.

[10] T. Geiger, B. Kästle, F. L. Gratani et al., “Two small (p)ppGpp synthases in Staphylococcus aureus mediate tolerance against cell envelope stress conditions,” *Journal of Bacteriology*, vol. 196, no. 4, pp. 894–902, 2014.

[11] A. S. Nuxoll, S. M. Halouska, M. R. Sadykov et al., “CcpA regulates arginine biosynthesis in Staphylococcus aureus through repression of proline catabolism,” *PLoS pathogens*, vol. 8, no. 11, e1003033, 2012.

[12] N. Philippi, D. Walter, R. Schlatter et al., “Modeling system states in liver cells: Survival, apoptosis and their modifications in response to viral infection,” *BMC systems biology*, vol. 3, p. 97, 2009.

[13] A. Di Cara, A. Garg, G. de Micheli et al., “Dynamic simulation of regulatory networks using SQUAD,” *BMC bioinformatics*, vol. 8, p. 462, 2007.

[14] K. Pohl, P. Francois, L. Stenz et al., “CodY in Staphylococcus aureus: A regulatory link between metabolism and virulence gene expression,” *Journal of Bacteriology*, vol. 191, no. 9, pp. 2953–2963, 2009.

[15] C. D. Majerczyk, M. R. Sadykov, T. T. Luong et al., “Staphylococcus aureus CodY negatively regulates virulence gene expression,” *Journal of Bacteriology*, vol. 190, no. 7, pp. 2257–2265, 2008.

[16] S. M. K. Schoenfelder, G. Marincola, T. Geiger et al., “Methionine biosynthesis in Staphylococcus aureus is tightly controlled by a hierarchical network involving an initiator tRNA-specific T-box riboswitch,” *PLoS pathogens*, vol. 9, no. 9, e1003606, 2013.

[17] T. Lei, *Characterization of a novel essential protein Gcp in Staphylococcus aureus,* Dissertation, University of Minnesota, 2012.

[18] C. Kohler, C. von Eiff, M. Liebeke et al., “A defect in menadione biosynthesis induces global changes in gene expression in Staphylococcus aureus,” *Journal of Bacteriology*, vol. 190, no. 19, pp. 6351–6364, 2008.

[19] M. A. Marra, S. J. M. Jones, C. R. Astell et al., “The Genome sequence of the SARS-associated coronavirus,” *Science (New York, N.Y.)*, vol. 300, no. 5624, pp. 1399–1404, 2003.

[20] K. L. Anderson, C. Roberts, T. Disz et al., “Characterization of the Staphylococcus aureus heat shock, cold shock, stringent, and SOS responses and their effects on log-phase mRNA turnover,” *Journal of Bacteriology*, vol. 188, no. 19, pp. 6739–6756, 2006.

[21] C. B. Coutinho and L. G.E.O. Nutini, “Correlation Between the Essential Amino-acid Requirements of Staphylococcus aureus, their Phage Types and Antibiotic Patterns,” *Nature*, vol. 198, no. 4882, pp. 812–813, 1963.

[22] R. A. Lincoln, J. A. Leigh, and N. C. Jones, “The amino acid requirements of Staphylococcus aureus isolated from cases of bovine mastitis,” *Veterinary Microbiology*, vol. 45, 2-3, pp. 275–279, 1995.

[23] R. Mah, D. Fung, and S. Morse, “Nutritional Requirements of Staphylococcus aureus S-6,” *Applied Microbiology*, vol. 15, no. 4, pp. 866–870, 1967.

[24] Gale E.F. and Rodwell A.W., “The assimilation of amino-acids by bacteria; 7. The nature of resistance to penicillin in Staphylococcus aureus,” *Journal of general microbiology*, vol. 3, no. 1, pp. 127–142, 1949.

[25] M. Kuroda, T. Ohta, I. Uchiyama et al., “Whole genome sequencing of meticillin-resistant Staphylococcus aureus,” *The Lancet*, vol. 357, no. 9264, pp. 1225–1240, 2001.

[26] Kloos W.E. and Schleifer K.H., “Genus IV. Staphylococcus Rosenbach 1884,” in *Bergey's manual of systematic bacteriology. First edition, vol. 2*: *Gram-positive Bacteria other than Actinomycetes,* Sneath P.H.A., Mair N.S., Sharpe M.E. et al., Eds., pp. 1013–1035, Williams & Wilkins, Baltimore, Md., 1986.

[27] R. A. Proctor, “Role of folate antagonists in the treatment of methicillin-resistant Staphylococcus aureus infection,” *Clinical infectious diseases : an official publication of the Infectious Diseases Society of America*, vol. 46, no. 4, pp. 584–593, 2008.

[28] J. G. Bartlett, “Antibiotic-Associated Pseudomembranous Colitis Due to Toxin-Producing Clostridia,”.

[29] S. Herbert, A.-K. Ziebandt, K. Ohlsen et al., “Repair of global regulators in Staphylococcus aureus 8325 and comparative analysis with other clinical isolates,” *Infection and immunity*, vol. 78, no. 6, pp. 2877–2889, 2010.

[30] S. Fuchs, J. Pané-Farré, C. Kohler et al., “Anaerobic gene expression in Staphylococcus aureus,” *Journal of Bacteriology*, vol. 189, no. 11, pp. 4275–4289, 2007.

[31] M. J. Pucci, J. A. Thanassi, H. T. Ho et al., “Staphylococcus haemolyticus contains two D-glutamic acid biosynthetic activities, a glutamate racemase and a D-amino acid transaminase,” *Journal of Bacteriology*, vol. 177, no. 2, pp. 336–342, 1995.

[32] A. G. Moat, J. W. Foster, and M. P. Spector, *Microbial physiology*, Wiley-Liss, New York, 2002.

[33] M. J. Kobylarz, *Siderophore-mediated iron metabolism in staphylococcus aureus,* Dissertation, The university of british columbia, 02.2016.

[34] E. Bore, S. Langsrud, Ø. Langsrud et al., “Acid-shock responses in Staphylococcus aureus investigated by global gene expression analysis,” *Microbiology (Reading, England)*, vol. 153, Pt 7, pp. 2289–2303, 2007.

[35] K. C. Strasters and K. C. Winkler, “Carbohydrate metabolism of staphylococcus aureus,” *Journal of general microbiology*, vol. 33, pp. 213–229, 1963.

[36] Y. Tanaka, K. Morikawa, Y. Ohki et al., “Structural and mutational analyses of Drp35 from Staphylococcus aureus: A possible mechanism for its lactonase activity,” *The Journal of biological chemistry*, vol. 282, no. 8, pp. 5770–5780, 2007.

[37] C. D. Smith and P. A. Pattee, “Biochemical and Genetic Analysis of Isoleucine and Valine Biosynthesis in Staphylococcus aureus,” *Journal of Bacteriology*, vol. 93, no. 6, pp. 1832–1838, 1967.

[38] J. M. Berg, J. L. Tymoczko, and L. Stryer, *Biochemie*, Spektrum Akad. Verl., Heidelberg, 2003.

[39] E. M. Panina, A. G. Vitreschak, A. A. Mironov et al., “Regulation of biosynthesis and transport of aromatic amino acids in low-GC Gram-positive bacteria,” *FEMS Microbiology Letters*, vol. 222, no. 2, pp. 211–220, 2003.

[40] M. D. Valentino, L. Foulston, A. Sadaka et al., “Genes contributing to Staphylococcus aureus fitness in abscess- and infection-related ecologies,” *mBio*, vol. 5, no. 5, e01729-14, 2014.

[41] B. M. Benton, J. P. Zhang, S. Bond et al., “Large-scale identification of genes required for full virulence of Staphylococcus aureus,” *Journal of Bacteriology*, vol. 186, no. 24, pp. 8478–8489, 2004.

[42] D. A. Rodionov, “Regulation of lysine biosynthesis and transport genes in bacteria: Yet another RNA riboswitch?,” *Nucleic acids research*, vol. 31, no. 23, pp. 6748–6757, 2003.

[43] A. Nakamura, M. Yao, S. Chimnaronk et al., “Ammonia channel couples glutaminase with transamidase reactions in GatCAB,” *Science (New York, N.Y.)*, vol. 312, no. 5782, pp. 1954–1958, 2006.

[44] S. R. Mladenova, K. R. Stein, L. Bartlett et al., “Relaxed tRNA specificity of the Staphylococcus aureus aspartyl-tRNA synthetase enables RNA-dependent asparagine biosynthesis,” *FEBS letters*, vol. 588, no. 9, pp. 1808–1812, 2014.

[45] R. M. Corrigan, J. C. Abbott, H. Burhenne et al., “c-di-AMP is a new second messenger in Staphylococcus aureus with a role in controlling cell size and envelope stress,” *PLoS pathogens*, vol. 7, no. 9, e1002217, 2011.

[46] R. M. Corrigan, L. Bowman, A. R. Willis et al., “Cross-talk between two nucleotide-signaling pathways in Staphylococcus aureus,” *The Journal of biological chemistry*, vol. 290, no. 9, pp. 5826–5839, 2015.

[47] A. Proctor and W. Kloos, “Tryptophan Biosynthetic Enzymes of Staphylococcus aureus,” *Journal of Bacteriology*, vol. 1973, no. 1, pp. 169–177, 144.

[48] C. O'Connell, P. A. Pattee, and T. J. Foster, “Sequence and mapping of the aroA gene of Staphylococcus aureus 8325-4,” *Journal of general microbiology*, vol. 139, no. 7, pp. 1449–1460, 1993.

[49] T. T. Luong, K. Sau, C. Roux et al., “Staphylococcus aureus ClpC divergently regulates capsule via sae and codY in strain newman but activates capsule via codY in strain UAMS-1 and in strain Newman with repaired saeS,” *Journal of Bacteriology*, vol. 193, no. 3, pp. 686–694, 2011.

[50] J. K. Lithgow, E. J. Hayhurst, G. Cohen et al., “Role of a Cysteine Synthase in Staphylococcus aureus,” *Journal of Bacteriology*, vol. 186, no. 6, pp. 1579–1590, 2004.

[51] B. Krismer, M. Liebeke, D. Janek et al., “Nutrient limitation governs Staphylococcus aureus metabolism and niche adaptation in the human nose,” *PLoS pathogens*, vol. 10, no. 1, e1003862, 2014.

[52] D. A. Rodionov, A. G. Vitreschak, A. A. Mironov et al., “Comparative genomics of the methionine metabolism in Gram-positive bacteria: A variety of regulatory systems,” *Nucleic acids research*, vol. 32, no. 11, pp. 3340–3353, 2004.

[53] G. A. Somerville and R. A. Proctor, “At the crossroads of bacterial metabolism and virulence factor synthesis in Staphylococci,” *Microbiology and molecular biology reviews : MMBR*, vol. 73, no. 2, pp. 233–248, 2009.

[54] M. Liebeke, K. Dörries, D. Zühlke et al., “A metabolomics and proteomics study of the adaptation of Staphylococcus aureus to glucose starvation,” *Molecular bioSystems*, vol. 7, no. 4, pp. 1241–1253, 2011.

[55] Y. Zhu, R. Nandakumar, M. R. Sadykov et al., “RpiR homologues may link Staphylococcus aureus RNAIII synthesis and pentose phosphate pathway regulation,” *Journal of Bacteriology*, vol. 193, no. 22, pp. 6187–6196, 2011.

[56] W. E. Kloos and P. A. Pattee, “A biochemical characterization of histidine-dependant mutants of staphylococcus aureus,” *Journal of general microbiology*, vol. 39, pp. 185–194, 1965.

[57] J. Leiba, T. Hartmann, M.-E. Cluzel et al., “A novel mode of regulation of the Staphylococcus aureus catabolite control protein A (CcpA) mediated by Stk1 protein phosphorylation,” *The Journal of biological chemistry*, vol. 287, no. 52, pp. 43607–43619, 2012.

[58] B. R. Boles and A. R. Horswill, “Agr-mediated dispersal of Staphylococcus aureus biofilms,” *PLoS pathogens*, vol. 4, no. 4, e1000052, 2008.

[59] T. L. Campbell, J. Henderson, D. E. Heinrichs et al., “The yjeQ gene is required for virulence of Staphylococcus aureus,” *Infection and immunity*, vol. 74, no. 8, pp. 4918–4921, 2006.

[60] V. Dengler, N. McCallum, P. Kiefer et al., “Mutation in the C-di-AMP cyclase dacA affects fitness and resistance of methicillin resistant Staphylococcus aureus,” *PloS one*, vol. 8, no. 8, e73512, 2013.

[61] S. Karl and T. Dandekar, “Jimena: efficient computing and system state identification for genetic regulatory networks,” *BMC Bioinformatics*, vol. 14, no. 1, p. 306, 2013.
