# Supplementary figures and images for: Modeling of stringent-response reflects nutrient stress induced growth impairment and essential amino acids in different Staphylococcus aureus mutants
Source: Sci Rep. 2021 May 6;11:9651. doi: 10.1038/s41598-021-88646-1 (PMC8102509; doi:10.1038/s41598-021-88646-1)

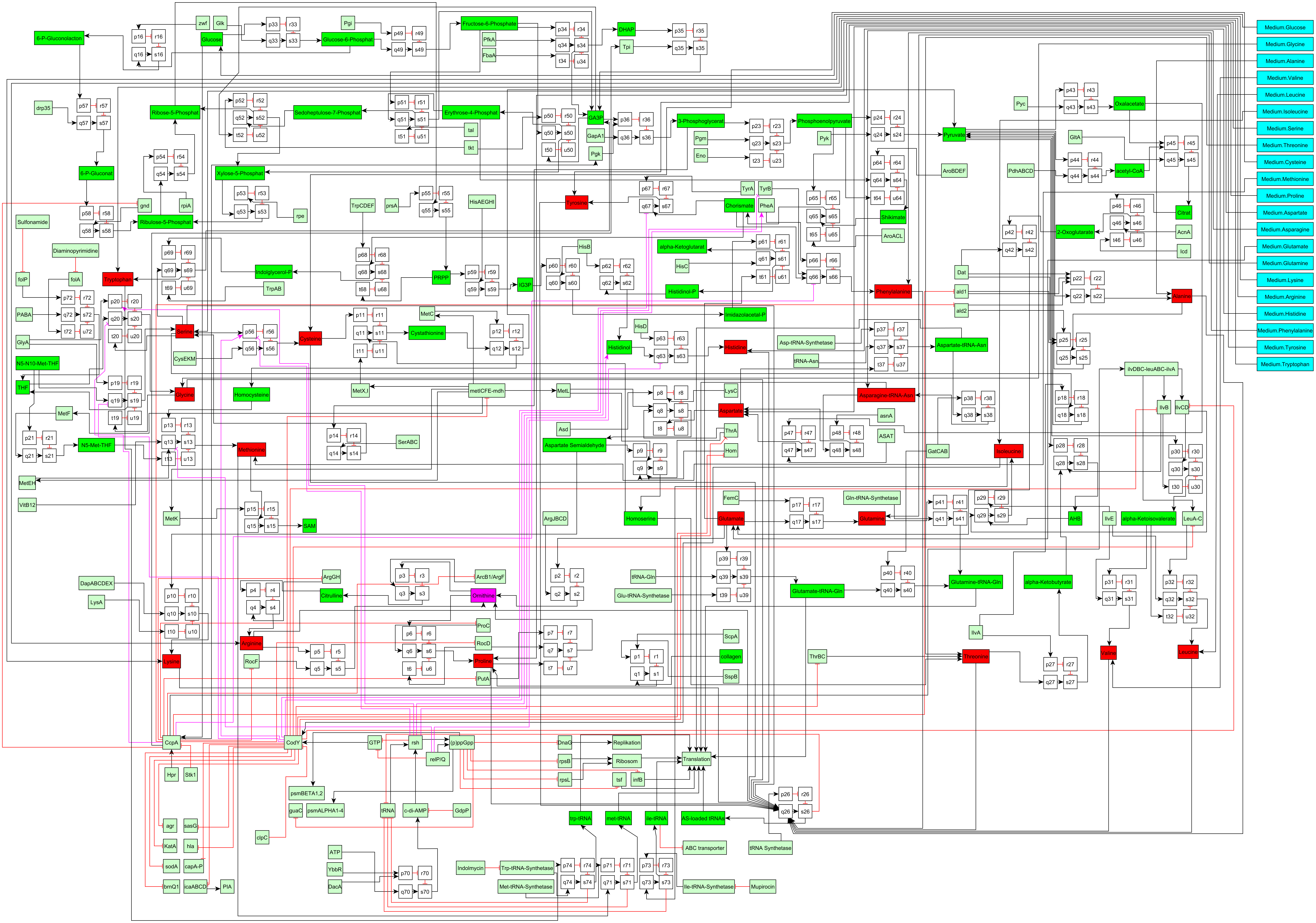

Supplement: Supplementary file 2 — Supplementary Information 2. [file 41598_2021_88646_MOESM2_ESM.jpg]
